# Photochemistry of glyoxylate embedded in sodium chloride clusters, a laboratory model for tropospheric sea-salt aerosols†
†Electronic supplementary information (ESI) available. See DOI: 10.1039/c8cp00399h


**DOI:** 10.1039/c8cp00399h

**Published:** 2018-02-28

**Authors:** Nina K. Bersenkowitsch, Milan Ončák, Christian van der Linde, Andreas Herburger, Martin K. Beyer

**Affiliations:** a Institut für Ionenphysik und Angewandte Physik , Universität Innsbruck , Technikerstraße 25 , 6020 Innsbruck , Austria . Email: milan.oncak@uibk.ac.at ; Email: martin.beyer@uibk.ac.at

## Abstract

Although marine aerosols undergo extensive photochemical processing in the troposphere, a molecular level understanding of the elementary steps involved in these complex reaction sequences is still missing. As a defined laboratory model system, the photodissociation of sea salt clusters doped with glyoxylate, [Na_*n*_Cl_*n*–2_(C_2_HO_3_)]^+^, *n* = 5–11, is studied by a combination of mass spectrometry, laser spectroscopy and *ab initio* calculations. Glyoxylate acts as a chromophore, absorbing light below 400 nm *via* two absorption bands centered at about 346 and 231 nm. Cluster fragmentation dominates, which corresponds to internal conversion of the excited state energy into vibrational modes of the electronic ground state and subsequent unimolecular dissociation. Photochemical dissociation pathways in electronically excited states include CO and HCO elimination, leading to [Na_*n*–*x*_Cl_*n–x*–2_HCOO]^+^ and [Na_*n*_Cl_*n*–2_COO˙]^+^ with typical quantum yields in the range of 1–3% and 5–10%, respectively, for *n* = 5. The latter species contains CO_2_˙^–^ stabilized by the salt environment. The comparison of different cluster sizes shows that the fragments containing a carbon dioxide radical anion appear in a broad spectral region of 310–380 nm. This suggests that the elusive CO_2_˙^–^ species may be formed by natural processes in the troposphere. Based on the photochemical cross sections obtained here, the photolysis lifetime of glyoxylate in a dry marine aerosol is estimated as 10 h. Quantum chemical calculations show that dissociation along the C–C bond in glyoxylic acid as well as glyoxylate embedded in the salt cluster occurs after reaching the S_1_/S_0_ conical intersection, while this conical intersection is absent in free glyoxylate ions.

## Abstract 2

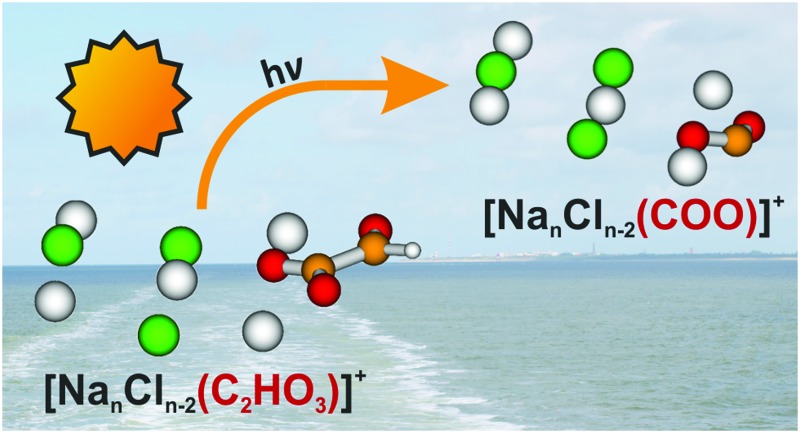
Although marine aerosols undergo extensive photochemical processing in the troposphere, a molecular level understanding of the elementary steps involved in these complex reaction sequences is still missing.

## Introduction

Marine aerosols^1^–^4^ are composed of a complex mixture of inorganic as well as organic components. Besides sodium chloride and water as the main constituents, hydrocarbons such as lipids, acids, humic substances, and saccharides play an essential role in the chemistry of marine aerosols.^3^,^5^–^8^ Sea spray aerosols are produced by the mechanical disruption of the ocean surface,^9^ with the size of the released droplets depending strongly on the wind speed.^10^,^11^ Gas to particle conversion processes like nucleation and condensation form secondary sea salt aerosols including organic as well as non-organic constituents, *e.g.* sulphate.^12^

As the ocean covers more than 70% of the Earth's surface, this type of natural aerosol is highly relevant for atmospheric processes. The complexity of these processes is a challenge for numerical climate models.^13^,^14^ Aerosols provide condensation nuclei for cloud droplets and sea salt particles cannot only backscatter,^2^ but also absorb solar radiation.^15^,^16^ Sea-salt aerosols exhibit a rich chemistry with atmospheric trace gases^17^ and complex photochemical reactions.^18^,^19^ Photochemical processing of organic matter^9^,^19^,^20^ is initiated by sunlight, and highly reactive species such as OH˙ can be produced.^21^,^22^

Gas-phase sodium chloride clusters^23^ are well-established model systems for salt surfaces.^24^ In the present study, sodium chloride is used as a basis for modeling photochemical reactions on salt clusters. The clusters are doped with glyoxylic acid,^25^–^27^ one of the most abundant 2-oxocarboxylic acids^25^ in organic aerosols in environments like the North China Plain^28^ or marine regions^29^,^30^ such as the eastern North Pacific. The neutral molecule absorbs in the actinic region with *λ* > 290 nm, the wavelength range of solar radiation that reaches the troposphere.^26^ Since in aqueous solution at neutral pH the *gem*-diol form HC(OH)_2_COO^–^ dominates,^31^ we cannot rule out that glyoxylate in wet sea-salt aerosols may also be present as *gem*-diol. Our study using dry HCOCOO^–^ thus represents only the first step towards a comprehensive understanding of glyoxylate photochemistry in this environment.

In the gas phase, the major products of glyoxylic acid photodissociation as well as its thermal decomposition are CO_2_ and CH_2_O, with minor contributions of CO and H_2_.^26^ Quantum chemical calculations suggested high barriers for the lowest energy unimolecular decomposition pathway, in the range of 200 kJ mol^–1^.^32^ Hydration with a single water molecule leads to an only mild reduction of the barrier for H_2_ formation, from 214 kJ mol^–1^ to 181 kJ mol^–1^.^33^ Under experimental conditions in the gas or liquid phase, however, bimolecular collisions may be involved, enabling additional reaction pathways. Vaida and co-workers showed by vibrational spectroscopy that glyoxylic acid is also present in its geminal diol form.^34^ They also showed that vibrational overtone excitation of the O–H mode with four or five vibrational quanta leads to decomposition of glyoxylic acid if the hydrogen atom is oriented to form an intramolecular hydrogen bond.^35^ The isomerization reactions involved in formation of this intramolecular hydrogen bond can be induced by near-infrared light, as shown in argon matrices by Olbert-Majkut *et al.*^36^ In aqueous solution, Guzman and co-workers identified a number of photolysis products, including CO_2_, CO, formic acid, oxalic acid, and tartaric acid.^25^ They propose the homolytic cleavage of the C–C bond as the first step in the reaction cascade, followed by a series of bimolecular reactions. Related studies can be found for pyruvic acid.^37^–^39^

Photocatalytic oxidation of glyoxylate adsorbed on quantum-sized ZnO was observed by Hoffmann and co-workers.^40^ Ekström and McQuillan reported the coordination of glyoxylate on TiO_2_ in aqueous solution in its hydrated form, and the formation of adsorbed oxalate upon near-UV irradiation.^41^ In the gas–solid system, glyoxylate is adsorbed in several ways on TiO_2_, reported by Ho *et al.*^42^ Thermal decomposition results in CO, CO_2_, adsorbed formate and methoxy groups, while photodissociation at 350–450 nm leads to CO_2_, carbonate and formate. All these surface studies are interpreted in terms of photocatalytic activity of the support, while direct photolysis of *e.g.* the C–C bond in glyoxylate was not considered.

Here we address the photochemistry of cationic sodium chloride clusters doped with a glyoxylate anion. Photodissociation cross sections are measured, and the photodissociation products are identified by mass spectrometry. Quantum chemical calculations of ground and first excited state provide a molecular level understanding of the observed photodissociation pathways. Comparison of the situation of the ion embedded in the cluster with the bare ion as well as the neutral molecule in the gas phase reveals the influence of the ionic environment.

## Experimental and theoretical details

The experimental setup consists of a Bruker Apex Qe Fourier transform ion cyclotron resonance (FT-ICR) mass spectrometer interfaced to a tunable optical parametric oscillator (EKSPLA NT342B) as described in detail before.^43^ Briefly, cluster ions Na_*n*+1_Cl_*n*–1_(C_2_HO_3_)^+^ are generated by electrospray ionization and transferred to a hexapole collision cell where they are thermalized in collisions with argon at room temperature. After injection into the ICR cell, the ion of interest is mass selected by resonant excitation of unwanted ions.

Tunable laser light is allowed into the ICR cell for a defined period of time controlled by a mechanical shutter. Due to the long irradiation times of up to 20 s used in the present study, also very weak photodissociation cross sections can be determined quantitatively. For the calculation of the total photodissociation cross section, also the fragmentation occurring due to black body infrared radiation dissociation (BIRD)^44^ has to be taken into account, eqn (1).1
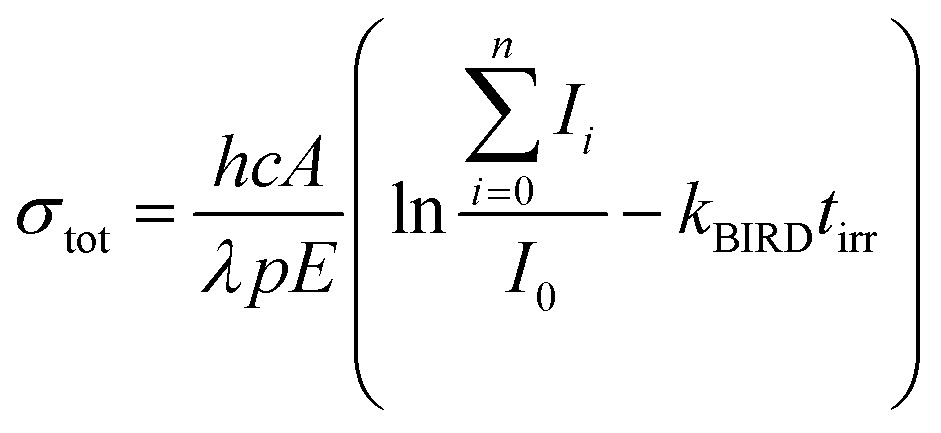



*I*_0_ represents the intensity of the precursor ion after laser irradiation, *I*_*i*_ the corresponding intensities of fragment *i*, *h* Planck's constant, *c* the speed of light, *A* the area illuminated by the laser beam, *λ* the wavelength, *p* the number of laser pulses, *E* the energy of a laser pulse, *t*_irr_ the irradiation time of the precursor ion and *k*_BIRD_ the rate constant for BIRD. This quantity can be measured independently *via*eqn (2) where the precursor ion is stored for different times and exposed to the room temperature blackbody radiation. Since the dissociation due to BIRD follows an exponential law, the fit gives the rate constant *k*_BIRD_.

2 *I*_0_(*t*) = *I*_0_e^–*k*_BIRD_*t*_irr_^

Partial cross sections of the individual fragments *σ*_*i*_ are calculated *via*eqn (3):3
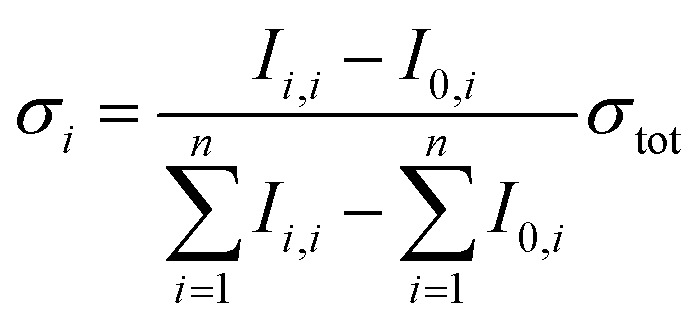
where *I*_*i*,*i*_ is the intensity of the fragment after irradiation of the precursor ion with UV light and *I*_0,*i*_ the intensity of the fragment contribution due to BIRD.

The main sources of error of the absolute cross section are the measured rate constant *k*_BIRD_ and the photon flux inside the ICR cell. A peak at Δ*m*/*z* = 0.17 was present in small amounts in most experiments, which was too close to be ejected without exciting the ion of interest. This peak was assigned, and for stoichiometric reasons its photofragments do not interfere with the major photofragments of the ion of interest, see Fig. S1 (ESI†).

Pulse energies in UV fluctuate significantly since UV photons are generated in four stages of non-linear optics in the OPO system. Together with uncertainties in the alignment of the laser beam and beam profile in the ICR cell, located at a distance of 3 m from the laser system, the absolute cross sections reported here are estimated to be accurate within 30%.

All chemicals were purchased from Sigma Aldrich with a purity of at least 98%. Isotopically enriched Na^35^Cl is used for the studies, as mass spectra become significantly complicated with increasing cluster size due to the two stable isotopes of Cl. In all measurements, a 1 : 1 mixture of CH_3_OH/H_2_O (HPLC grade) was used as solvent. The measurements were performed with a NaCl concentration of 5 mmol L^–1^ and 1 mmol L^–1^ glyoxylic acid.

Structure and photochemistry of the clusters were also explored using methods of theoretical chemistry. Initial structures were taken from the Cambridge Cluster Database^45^ for [Na_*n*_Cl_*n*+1_]^–^ clusters and modified to [Na_*n*+1_Cl_*n*_]^+^. For each cluster size and for each position of the Cl^–^ ion, we replaced the Cl^–^ ion by the C_2_HO_3_^–^ anion and optimized the cluster at the respective level of theory. For *n* = 5, further isomers with different O–C–C–O dihedral angle were created starting from the respective minima. Several structures were also created by incorporating the ion into the cluster or on its surface. Initial structures for clusters after glyoxylate dissociation were built from the optimized clusters. Cluster optimization was performed at the B3LYP/def2TZVP and B3LYP/6-31+g* level of theory for smaller and larger systems, respectively, with the dispersion correction (D2) as introduced by Grimme.^46^ We note that this correction changes both the relative stability of clusters and their structure (see Table S1, ESI†).

Excited states of clusters were calculated at the EOM-CCSD level. The TDDFT method with two tested hybrid functionals, BHandHLYP and CAM-B3LYP, predicted artificial charge transfer states when Cl^–^ ions were present in the cluster (see Table S2 and the corresponding discussion, ESI†), and could thus not be used. The excited states character was analyzed for the C_2_HO_3_^–^ ion by calculation of natural transition orbitals at the TD-BHandHLYP level.^47^ Note that for calculation of excited states higher than S_1_ in C_2_HO_3_^–^, no diffuse functions were deliberately added; when diffuse functions are used, *e.g.* within the aug-cc-pVXZ basis set series, pre-dissociation states start appearing below 5 eV (at the B3LYP+D2/def2TZVP level of theory, the vertical ionization potential of C_2_HO_3_^–^ in the gas phase is predicted to be 3.7 eV); these states disappear for glyoxylate in the salt environment. The shape of the C_2_HO_3_^–^ absorption spectra was modeled using the linearized reflection principle approximation.^48^,^49^ Scans in the excited states were performed at the complete active space – self-consistent field (CASSCF) level of theory with the active space of (6,6) and def2TZVP basis for C, O, H and the Stuttgart basis set^50^,^51^ for Na and Cl that do not participate in the excitation in order to speed up the calculations. Only two electronic states were included in the state average due to convergence issues when further low-lying excited states are treated. Multireference Configuration Interaction (MRCI) calculations with the same basis set were performed for structures optimized at the CASSCF level.

DFT and TDDFT calculations were done in the Gaussian software package,^52^ EOM-CCSD, CASSCF and MRCI calculations in the Molpro program.^53^

## Results and discussion

### Photodissociation spectrum of [Na_5_Cl_3_(C_2_HO_3_)]^+^ in the range of 225–400 nm

**Fig. 1:**
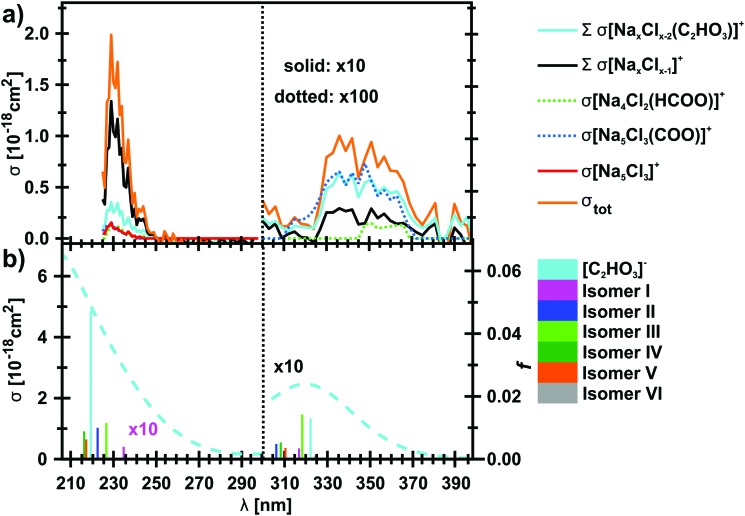
(a) Measured total photodissociation cross section of the [Na_5_Cl_3_(C_2_HO_3_)]^+^ cluster as well as the contribution of specific fragments in the 225–400 nm range. In the range of 300–400 nm, only the contribution of the most intense fragments is shown. (b) Calculated excitation energies and oscillator strengths *f* of C_2_HO_3_^–^ and several [Na_5_Cl_3_(C_2_HO_3_)]^+^ isomers (bars), modelled absorption spectrum of C_2_HO_3_^–^ using the linearized reflection principle (dashed line). Calculated at the EOM-CCSD/cc-pVDZ//B3LYP+D2/def2TZVP level of theory.

The photodissociation spectrum of the [Na_5_Cl_3_(C_2_HO_3_)]^+^ cluster was investigated experimentally in the range of 225–400 nm. The measured photodissociation cross section for [Na_5_Cl_3_(C_2_HO_3_)]^+^, including the contribution of the individual fragments, is shown in Fig. 1a. There is a strong absorption band at 230–250 nm with cross section on the order of 10^–18^ cm^2^, and a weaker band located at about 320–380 nm with cross sections below 10^–19^ cm^2^.

**Fig. 2:**
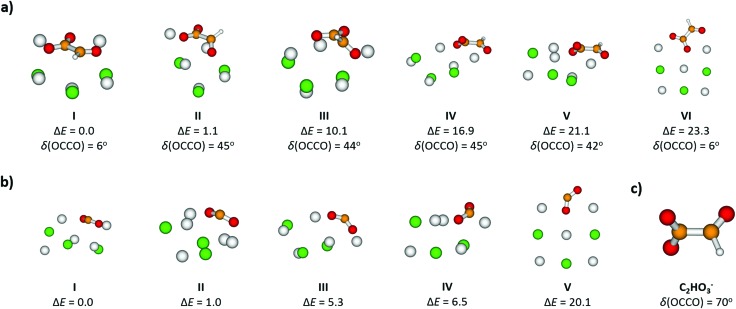
Structures, relative energies Δ*E* (in kJ mol^–1^) and dihedral angles *δ* (in degrees) of selected isomers of (a) [Na_5_Cl_3_(C_2_HO_3_)]^+^ and (b) [Na_5_Cl_3_(CO_2_)]^+^; (c) C_2_HO_3_^–^ ion is shown for comparison. Calculated at the B3LYP+D2/def2TZVP level of theory.

**Fig. 3:**
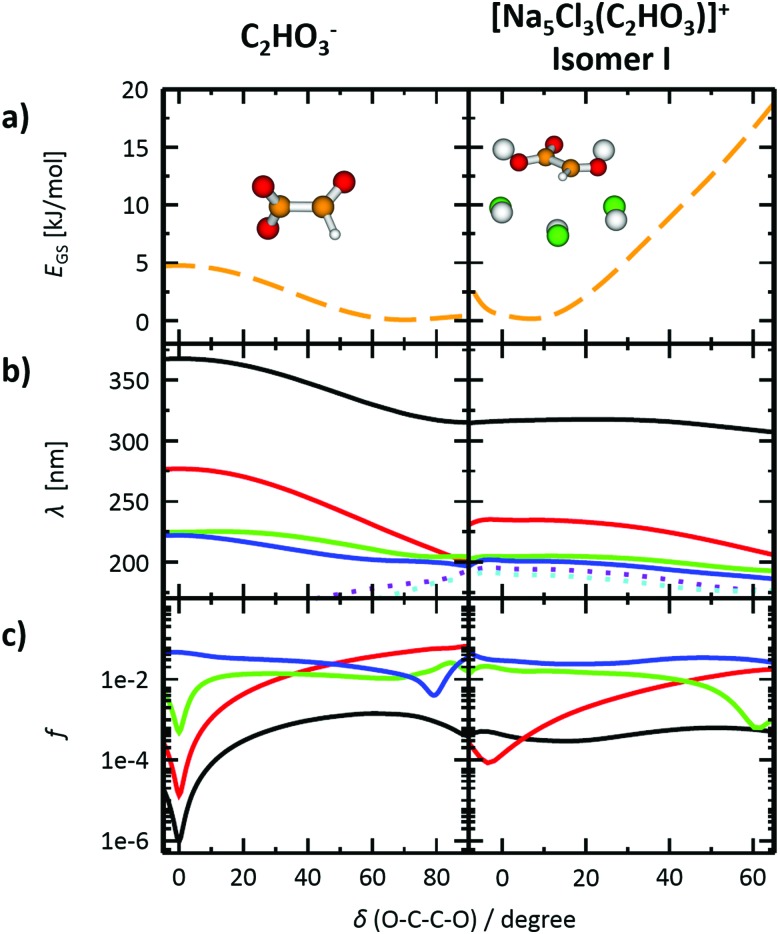
Photochemistry of C_2_HO_3_^–^ and [Na_5_Cl_3_(C_2_HO_3_)]^+^, isomer **I**. (a) Scan of the ground state energy *E*_GS_ in dependence on the O–C–C–O dihedral angle, calculated at the B3LYP+D2/def2TZVP level, with geometry fully optimized while constraining the dihedral angle value. (b) Excitation wavelengths *λ* of four lowest excited states calculated in the structure corresponding to part (a) (solid lines). Two further higher lying excited states are shown for reference (dotted lines). Calculated at the EOM-CCSD/cc-pVDZ level of theory. (c) Oscillator strengths *f* of the transitions shown in (b), employing the same color code for the electronic states.

To gain further insight into the absorption spectrum, theoretical calculations of excited states for different isomers of the cluster as well as for the isolated glyoxylate are shown in Fig. 1b, with the respective model systems provided in Fig. 2 and spectrum decomposition of two selected ions in Fig. 3 while scanning the O–C–C–O dihedral angle. In the C_2_HO_3_^–^ ion, the first absorption band is composed of only one transition of σ_2p,C–C_/π*_C–O_ character, while the second absorption band includes three different transitions of n/π_C–C_, n/π*_C–O_ and σ_2p,C–C_/π*_CO_2__ character, see orbitals in Fig. S3 (ESI†). Both excitation wavelength and transition dipole moment show strong dependence on the O–C–C–O dihedral angle *δ* (Fig. 3). In C_2_HO_3_^–^, there is a very low barrier for rotation along the dihedral angle, about 5 kJ mol^–1^ at the B3LYP+D2/def2TZVP level of theory, and a broad absorption spectrum can be expected. The width of the spectrum induced by the anharmonic effects, however, is neglected in the spectrum in Fig. 1 as it is simulated within the linearized reflection principle approximation.

For [Na_5_Cl_3_(C_2_HO_3_)]^+^, calculations show that there are several isomers that lie close in energy (Fig. 2a), differing in position of the glyoxylate ion within the cluster and in the value of the O–C–C–O dihedral angle. When the C_2_HO_3_^–^ ion interacts with the salt cluster, there is a slight shift in the excitation energies and the movement along the O–C–C–O angle becomes hindered as illustrated in the right-hand side of Fig. 3. For C_2_HO_3_^–^ embedded in the cluster, we can thus expect hindered rotation along the O–C–C–O dihedral angle and sharper bands in the absorption spectrum. However, only two absorption bands are still to be anticipated within the range of 200–400 nm. The oscillator strength of the second electronic transition depends sensitively on the interaction of the glyoxylate chromophore with the ionic environment.

Electronic transitions of selected [Na_5_Cl_3_(C_2_HO_3_)]^+^ isomers are shown in Fig. 1b. The position of the first excited state in the 300–325 nm region is similar for all studied isomers while the relative intensity of the respective absorption band will be markedly affected by changes in the O–C–C–O dihedral angle, see Fig. 3. At lower wavelengths of 200–230 nm, two groups of isomers can be distinguished with respect to their O–C–C–O dihedral angle *δ*, with the isomers with *δ* ∼ 45° (**II–V**) absorbing considerably more in the 210–230 nm region. Compared to the spectrum of C_2_HO_3_^–^, the intensity of the 220–240 nm absorption band is predicted to be markedly reduced, and a more structured spectrum can be expected.

The theoretical calculations reproduce the two absorption bands observed in the experiment. Quantitatively, the computationally predicted band positions are overestimated by about 0.2–0.3 eV. They are, however, very sensitive to the O–C–C–O dihedral angle value and can therefore vary considerably when thermal effects are accounted for. Another possible explanation of the deviation is the neglect of the zero-point energy correction for dissociative processes that would shift excitation energies to lower values.^54^ With respect to the spectrum intensity, the calculated oscillator strengths amount to absolute cross sections of the same order of magnitude as observed in the experiment, 10^–19^ cm^2^ and 10^–18^ cm^2^ for first and second absorption band, respectively. The actual values, however, will be again considerably affected by isomer distribution and thermal effects.

### Photodissociation products of [Na_5_Cl_3_(C_2_HO_3_)]^+^ in the range of 225–400 nm

In Table 1, the observed photofragmentation channels (4)–(13) as well as the possible dissociation channels of the glyoxylate ion (1) and (2) are summarized together with the calculated dissociation energy. The dominant charged fragments are stoichiometric clusters, *i.e.* Na_*x*_Cl_*x*–1_^+^ or Na_*x*_Cl_*x*–2_(C_2_HO_3_)^+^. In the latter case, the glyoxylate moiety stays intact, and Na_5–*x*_Cl_5–*x*_ units are presumably lost. This implies the presence of a conical intersection^55^–^57^ between the ground state and the excited state(s) populated after excitation, which is required for internal conversion. At longer wavelengths, this is the preferred pathway, while at shorter wavelength, formation of Na_*x*_Cl_*x*–1_^+^ dominates. Again, stoichiometric Na_5–*x*_Cl_4–*x*_(C_2_HO_3_) clusters may dissociate, reactions (5a)–(7a), but further decomposition into Na_5–*x*_Cl_4–*x*_(HCOO) and CO, reactions (5b)–(7b), is almost isoenergetic, and sometimes even energetically preferred. The latter scenario, however, requires photochemical rearrangements on the excited state potential energy surface. Strong evidence that this photochemical rearrangement is possible is provided by the Na_*x*_Cl_*x*–2_(HCOO)^+^ fragments, reactions (10)–(12), which are weak but unambiguously identified.

**Table 1:** Reaction energies Δ*E* of various dissociation reactions of C_2_HO_3_^–^ and [Na_5_Cl_3_(C_2_HO_3_)]^+^ ions calculated at the B3LYP+D2/def2TZVP level of theory. Reactions marked with ‘*’ were calculated at the B3LYP+D2/def2TZVP//B3LYP+D2/6-31+g* level, with zero point energy corrections evaluated at the lower level of theory. The C_2_HO_3_ radical in reaction (13) is predicted to be unstable and dissociate into CO_2_ and CHO. With the exception of reactions (1–3), all reactions included in the table were observed in our experiment

No.	Reactant	Products	Δ*E* [eV]
(1)	C_2_HO_3_^–^	CHO^–^ + CO_2_	2.30
(2)		CO_2_˙^–^ + CHO˙	3.05
(3)	[Na_5_Cl_3_(C_2_HO_3_)]^+^	[Na_5_Cl_3_(CHO)]^+^ + CO_2_	2.43
(4)		[Na_5_Cl_3_(CO_2_)]˙^+^ + CHO˙	3.47
(5a)		Na_2_Cl^+^ + Na_3_Cl_2_(C_2_HO_3_)	2.52
(5b)		Na_2_Cl^+^ + Na_3_Cl_2_HCOO + CO	2.69
(6a)		Na_3_Cl_2_^+^ + Na_2_Cl(C_2_HO_3_)	3.00
(6b)		Na_3_Cl_2_^+^ + Na_2_ClHCOO + CO	3.01
(7a)		Na_4_Cl_3_^+^ + Na(C_2_HO_3_)	2.51
(7b)		Na_4_Cl_3_^+^ + NaHCOO + CO	2.45
(8)		[Na_2_(C_2_HO_3_)]^+^ + Na_3_Cl_3_	2.48
(9)		[Na_3_Cl(C_2_HO_3_)]^+^ + Na_2_Cl_2_	2.48
(10a)		[Na_3_Cl(HCOO)]^+^ + Na_2_Cl_2_·CO	2.31
(10b)		[Na_3_Cl(HCOO)]^+^ + Na_2_Cl_2_ + CO	2.48
(11a)		[Na_4_Cl_2_(HCOO)]^+^ + NaCl·CO	2.21
(11b)		[Na_4_Cl_2_(HCOO)]^+^ + NaCl + CO	2.40
(12)		[Na_5_Cl_3_(HCOO)]^+^ + CO	0.40
(13)		[Na_5_Cl_3_]^+^ + [C_2_HO_3_ → CO_2_ + CHO]	5.04
(14)*	[Na_6_Cl_4_(C_2_HO_3_)]^+^	[Na_6_Cl_4_(CO_2_)]˙^+^ + CHO˙	3.34
(15)*	[Na_7_Cl_5_(C_2_HO_3_)]^+^	[Na_7_Cl_5_(CO_2_)]˙^+^ + CHO˙	3.40
(16)*	[Na_8_Cl_6_(C_2_HO_3_)]^+^	[Na_8_Cl_6_(CO_2_)]˙^+^ + CHO˙	3.19
(17)*	[Na_9_Cl_7_(C_2_HO_3_)]^+^	[Na_9_Cl_7_(CO_2_)]˙^+^ + CHO˙	3.24
(18)*	[Na_10_Cl_8_(C_2_HO_3_)]^+^	[Na_10_Cl_8_(CO_2_)]˙^+^ + CHO˙	3.29
(19)*	[Na_11_Cl_9_(C_2_HO_3_)]^+^	[Na_11_Cl_9_(CO_2_)]˙^+^ + CHO˙	3.53

The final proof for excited state photochemistry is provided by the [Na_5_Cl_3_(CO_2_)]˙^+^ fragment, a carbon dioxide anion radical embedded in the salt cluster produced in reaction (4), along with the elimination of the formyl radical HCO˙,^58^ with a calculated reaction energy of 3.47 eV. For comparison, CO_2_ dissociation along reaction (3) is energetically more favorable with 2.43 eV, and might be expected to be a more efficient reaction channel in the ground state. This fragment, however, is not observed over the studied wavelength range. We note that the situation is similar for the photochemistry of the isolated glyoxylate anion, where the dissociation into CO_2_˙^–^ and HCO˙ is 0.75 eV higher in energy than the dissociation into CO_2_ and HCO^–^, see reactions (1) and (2) in Table 1. Collision induced dissociation in the hexapole collision cell of the APEX Qe instrument confirms that, in the electronic ground state, the clusters dissociate by loss of stoichiometric cluster fragments, without C–C bond cleavage.

Formation of CO_2_˙^–^ embedded into the salt cluster involves breaking the C–C bond of glyoxylate (see Fig. 2b for calculated structures). The calculated reaction energy of equation (4) shows that the dissociation is plausible for the experimental wavelength range. This dissociation has also been observed in photochemical studies of isolated glyoxylic acid in the gas phase^26^ or in aqueous solution.^25^ As noted above, excitation into S_1_ involves the movement of an electron from σ_2p_ → π*, causing the weakening of the C–C bond, see Fig. S3 (ESI†) for molecular orbitals of isolated glyoxylate.

### Photodissociation dynamics of [Na_5_Cl_3_(C_2_HO_3_)]^+^ in the first excited state

**Fig. 4:**
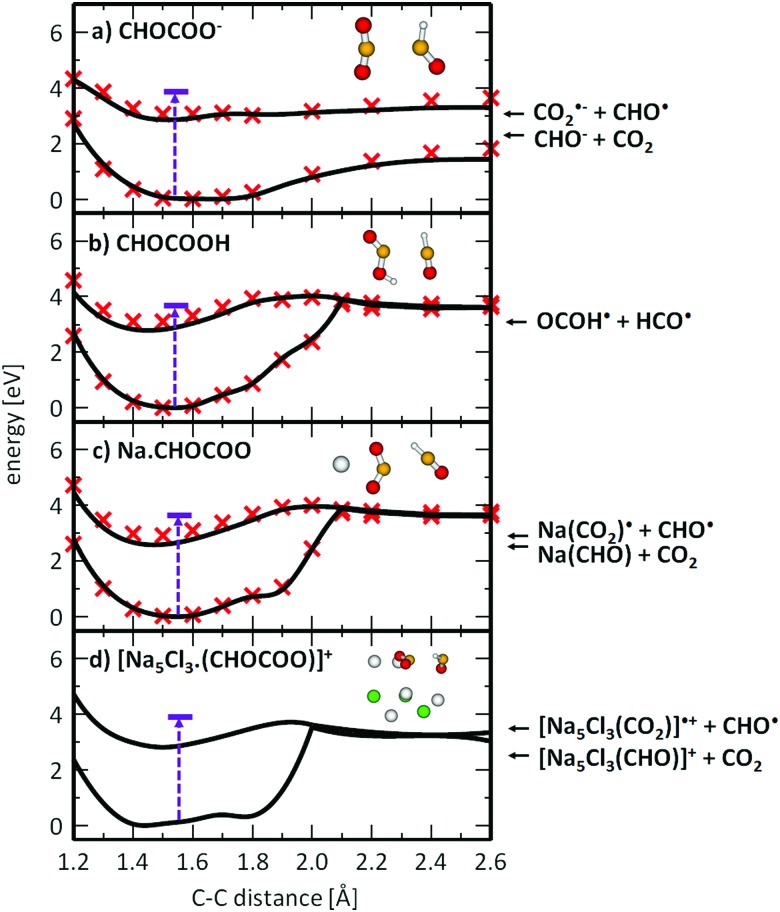
Photodissociation of glyoxylate anion (a) and glyoxylic acid (b) in the gas phase and glyoxylate anion in the salt cluster environment (c and d). Structures were optimized in the S_1_ state along the C–C dissociation coordinate at the CASSCF(6,6),SA2/def2-TZVP(C,H,O) + Stuttgart(Na,Cl) level of theory (black lines) and recalculated at the respective MRCI level (red crosses). Excitation energy in the ground state minimum structure (in violet) was calculated at the EOM-CCSD/def2-TZVP(C,H,O) + Stuttgart(Na,Cl) level in the structure optimized at the B3LYP+D2/def2-TZVP level, lying within 0.25 eV with respect to MRCI values for (a)–(c). Energetics of the exit channels was calculated at the B3LYP+D2/def2-TZVP level (arrows on the right-hand side).

To understand the photochemistry of glyoxylate and the influence of the salt environment in comparison with the neutral molecule, we studied photodissociation along the C–C coordinate in the S_1_ state using methods of theoretical chemistry. Fig. 4 illustrates the results for the glyoxylate anion and the neutral glyoxylic acid molecule in the gas phase, compared with glyoxylate interacting with a sodium ion and embedded in the salt cluster. The latter comparison is crucial since the NaC_2_HO_3_ system can still be treated on the higher level of theory (here MRCI) while the Na_5_Cl_3_C_2_HO_3_^+^ excited state is only tractable on the CASSCF level of theory. The excitation energy in the optimized ground state structure was added for reference.

When a glyoxylate ion in the gas phase is excited into S_1_ (Fig. 4a), it might reach a bound S_1_ minimum. However, the photoexcited anion has enough excess energy to directly dissociate along the C–C coordinate to form CO_2_ and CHO^–^* in its first excited state. The alternative CO_2_˙^–^ + CHO˙ dissociation channel lies higher in energy and becomes competitive only later along the dissociation coordinate, outside the region shown in Fig. 4a. Interestingly, there is no conical intersection, which renders internal conversion impossible for gas-phase glyoxylate within the investigated electronic states.

This picture changes dramatically when glyoxylate is protonated to form glyoxylic acid (Fig. 4b) or when it interacts with sodium ions (Fig. 4c and d). The photodissociation channel to produce CHO˙ and CO_2_˙^–^ or OCOH˙ becomes preferred. This can be rationalized by the effect of the positive charge, either the proton or sodium ions in the salt cluster, that stabilizes the charge on the CO_2_˙^–^ moiety. Along the reaction pathway, an S_1_/S_0_ conical intersection is reached at about *r*_C–C_ = 2.0 Å. In the [Na_5_Cl_3_(C_2_HO_3_)]^+^ cluster, the CASSCF calculations predict that the energy provided by the excitation into the S_1_ state suffices to directly reach this intersection. After funneling into the electronic ground state in the vicinity of the conical intersection, the glyoxylate anion might further dissociate, be formed again in a vibrationally hot ground electronic state, or the dissociating HCO˙ radical may react with CO_2_˙^–^ and transfer a hydrogen atom to form formate HCO_2_^–^ and CO. The barrier for such an H atom transfer in the ground state is calculated to be 1.93 eV and 2.75 eV for C_2_HO_3_^–^ and NaC_2_HO_3_, respectively, at the B3LYP+D2/def2TZVP level of theory. Both values are significantly smaller than the initial excitation energy, thus formate formation is energetically accessible. Due to the momentum gained along the dissociation coordinate, the main channel can be expected to be HOCO˙ and Na^+^·CO_2_˙^–^ formation for glyoxylic acid and Na(C_2_HO_3_), respectively. With the significantly larger number of degrees of freedom in Na_5_Cl_3_(C_2_HO_3_)^+^, however, the probability for energy redistribution increases, and internal conversion reaching the original structure may prevail, followed by statistical dissociation from the electronic ground state. This would explain the dominance of the stoichiometric fragments Na_*x*_Cl_*x*–2_(C_2_HO_3_)^+^ and Na_*x*_Cl_*x*–1_^+^. It may be noted that this photochemical reaction pathway does not require a photocatalyst, as implied in earlier studies of adsorbed glyoxylate.^40^–^42^

### The carbon dioxide radical anion and the Na_5_Cl_3_^+^ fragment

The most intriguing fragmentation channel is the dissociation of glyoxylate into the reactive HCO˙ radical and a carbon dioxide radical anion stabilized by the ionic environment of the salt cluster.^59^,^60^ In vacuum, the lifetime of CO_2_˙^–^ is only a few microseconds, because the free radical anion is metastable.^61^,^62^ However, interaction with the environment may stabilize CO_2_˙^–^, and it has been observed in liquid water,^63^ solvated by CO_2_ clusters^64^ or water molecules^65^–^70^ in gas phase, as well as in low-temperature rare gas matrices^71^,^72^ or in room-temperature alkali halides like NaBr, KCl, KBr, and KI.^73^,^74^ Due to the central role of CO_2_˙^–^ as an intermediate in a future electrochemical utilization of carbon dioxide,^75^ interest in the physicochemical properties of this elusive species has been rising steeply in the last decade.^60^ Our present results demonstrate that this fascinating radical anion is very likely also formed by natural processes in the troposphere.

It is important to note that the CO_2_˙^–^ fragment was found not only in the deeper UV in the wavelength range of 225–244 nm with no relevance for tropospheric chemical reactions (the corresponding light is completely absorbed in the stratosphere). The fragment was also measured in the actinic region of 310–370 nm, *i.e.* at wavelengths that reach the ground and thus are able to induce photochemical reactions in the troposphere.

The [Na_5_Cl_3_]˙^+^ fragment formed *via* reaction (13) is observed in the deeper UV up to 243 nm. Its formation requires a charge transfer reaction from glyoxylate to the positive charge centers, *i.e.* one or more sodium ions. This charge transfer followed by release and decomposition of the C_2_HO_3_˙ radical is calculated to be 5.04 eV endothermic. The C_2_HO_3_˙ radical is unstable and is predicted to dissociate into CO_2_ and CHO˙. This reaction may also proceed in two steps, *i.e.* CHO˙ dissociation followed by charge transfer with concomitant loss of CO_2_ from the intermediate Na_5_Cl_3_CO_2_^+^ cluster.

### Photodissociation cross section of [Na_*n*_Cl_*n*–2_(C_2_HO_3_)]^+^, *n* = 5–11, in the range of 300–400 nm

**Fig. 5:**
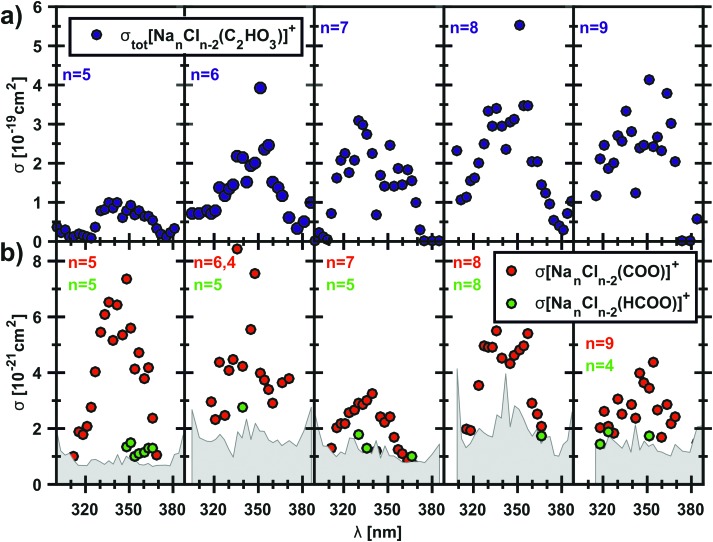
Measured total photodissociation cross sections of the [Na_*n*_Cl_*n*–2_(C_2_HO_3_)]^+^ cluster, *n* = 5–9, and the corresponding cross sections of the fragment with the carbon dioxide anion radical in the cluster.

To examine if the results for the [Na_5_Cl_3_(C_2_HO_3_)]^+^ ion might be extrapolated to sea salt aerosols, we repeated the experiment with clusters containing up to 11 sodium ions. All studied clusters exhibit the α-cleavage of the glyoxylate as a photodissociation channel, resulting in a sodium chloride cluster containing CO_2_˙^–^. Fig. 5 shows the total photodissociation cross section of the clusters with *n* = 5–9 and the very small cross sections of the corresponding CO_2_˙^–^ fragment down to 10^–21^ cm^–2^. The carbon dioxide anion fragments appear roughly in the wavelength range of 310–370 nm, which corresponds to 4.00–3.35 eV, in agreement with theoretically calculated energies that predict nearly constant dissociation energy of 3.2–3.5 eV in the investigated cluster size range, see reactions (14)–(19) in Table 1. For *n* = 5, the quantum yield lies typically in the range of 5–10%. The clusters with *n* = 6, 11 also show quantum yields up to 5.6% while the values for *n* = 7–10 are between 1–3% (see also Fig. S4, ESI†). This can be interpreted as competition of internal conversion with the C–C bond cleavage, as already mentioned above. Internal conversion will be favored with an increasing number of degrees of freedom in the larger clusters. Fragments containing HCOO^–^ that arise after a hydrogen transfer reaction are observed just above the noise level, with quantum yields of 1–3%. However, they are definitely identified as a photodissociation channel that competes with simple C–C bond cleavage. For clusters with *n* = 10, 11, the CO_2_˙^–^ fragment was observed only for two wavelengths (see Fig. S5, ESI†), due to the significantly increased noise level for the largest cluster sizes studied.

The measured photodissociation cross sections are used to estimate the photochemical lifetime of glyoxylate on the surface of sea-salt aerosols. Solar irradiance data was taken from terrestrial reference spectra for photovoltaic performance evaluation.^76^,^77^ Convolution of the partial photodissociation cross section for formation of the CO_2_˙^–^ and HCOO^–^ containing product clusters from the [Na_5_Cl_3_(C_2_HO_3_)]^+^ experiment with the global tilt spectrum^77^ results in a photochemical lifetime of 13.6 h for glyoxylate in the sea-salt environment, much shorter than the aqueous phase lifetime of 5 d for reaction with OH˙ radicals estimated by Guzman and co-workers.^25^ Since photochemical aging of sea-salt aerosols takes place on a similar time scale, photolysis of the C–C bond in glyoxylate may contribute to this process. However, under atmospheric conditions, glyoxylate may be present in its geminal diol form due to hydration.^31^ The study of hydration effects will be the next step towards a realistic modeling of glyoxylate photochemistry in the laboratory and by quantum chemistry.

## Conclusions

We investigated photodissociation of salt clusters doped with glyoxylate in the wavelength range of 225–400 nm. Internal conversion of the excitation energy results in loss of stoichiometric cluster fragments [NaCl]_*x*_ or [Na_*x*_Cl_*x*–1_(C_2_HO_3_)]. A genuinely photochemical product is the carbon dioxide radical anion CO_2_˙^–^ embedded in the salt environment. This radical is produced by photolysis of the C–C bond of glyoxylate, accompanied by the release of a neutral HCO˙ radical. The salt environment stabilizes CO_2_˙^–^. The radical anion was observed in the 300–400 nm wavelength range for all cluster sizes [Na_*n*_Cl_*n*–2_(C_2_HO_3_)]^+^, *n* = 5–11, suggesting that it is formed as a transient species in the troposphere. The photochemical lifetime calculated with typical solar irradiation data is in the range of 10 h, which renders the photodissociation of glyoxylate a potentially relevant reaction in the photochemical aging of sea-salt aerosols. Excited state calculations reveal that C–C bond cleavage takes places by surpassing a relatively small barrier along the excited state reaction coordinate and reaching the S_1_/S_0_ conical intersection. In the isolated glyoxylate anion in the gas phase, the S_0_ and S_1_ states are well separated, which prevents non-radiative relaxation to the electronic ground state. The electronic structure of gaseous neutral glyoxylic acid, however, is very similar to glyoxylate interacting with the salt environment. Repeating the calculations with NaC_2_HO_3_ reveals that the interaction of the deprotonated acid functional group with a single sodium ion is sufficient to restore the photochemical behavior of neutral glyoxylic acid.

## Conflicts of interest

There are no conflicts to declare.

## Supplementary Material

Supplementary information

## References

[cit1] O’Dowd C. D., de Leeuw G. (2007). Philos. Trans. A Math. Phys. Eng. Sci..

[cit2] Finlayson-Pitts B. J. and Pitts J. N., Chemistry of the Upper and Lower Atmosphere. Theory, Experiments, and Applications, Academic Press, San Diego, 2000.

[cit3] Gantt B., Meskhidze N. (2013). Atmos. Chem. Phys..

[cit4] Finlayson-Pitts B. J. (2003). Chem. Rev..

[cit5] Mkoma S. L., Kawamura K. (2013). Atmos. Chem. Phys..

[cit6] Kawamura K., Bikkina S. (2016). Atmos. Res..

[cit7] Tervahattu H. (2002). J. Geophys. Res..

[cit8] Chen J., Kawamura K., Liu C.-Q., Fu P. (2013). Atmos. Environ..

[cit9] Laskin A., Laskin J., Nizkorodov S. A. (2015). Chem. Rev..

[cit10] Zielinski T. (1997). Oceanologia.

[cit11] Goroch A. K., Fairall C. W., Davidson K. L. (1982). J. Appl. Meteorol..

[cit12] O’Dowd C. D., Smith M. H., Consterdine I. E., Lowe J. A. (1997). Atmos. Environ..

[cit13] Kanakidou M., Seinfeld J. H., Pandis S. N., Barnes I., Dentener F. J., Facchini M. C., van Dingenen R., Ervens B., Nenes A., Nielsen C. J., Swietlicki E., Putaud J. P., Balkanski Y., Fuzzi S., Horth J., Moortgat G. K., Winterhalter R., Myhre C. E. L., Tsigaridis K., Vignati E., Stephanou E. G., Wilson J. (2005). Atmos. Chem. Phys..

[cit14] Lohmann U., Feichter J. (2005). Atmos. Chem. Phys..

[cit15] Papadimas C. D., Hatzianastassiou N., Matsoukas C., Kanakidou M., Mihalopoulos N., Vardavas I. (2012). Atmos. Chem. Phys..

[cit16] Johnson B. T., Shine K. P., Forster P. M. (2004). Q. J. R. Meteorol. Soc..

[cit17] Beichert P., Finlayson-Pitts B. J. (1996). J. Phys. Chem..

[cit18] Richards N. K., Finlayson-Pitts B. J. (2012). Environ. Sci. Technol..

[cit19] George C., Ammann M., D’Anna B., Donaldson D. J., Nizkorodov S. A. (2015). Chem. Rev..

[cit20] Bernard F., Ciuraru R., Boreave A., George C. (2016). Environ. Sci. Technol..

[cit21] Zhou X., Davis A. J., Kieber D. J., Keene W. C., Maben J. R., Maring H., Dahl E. E., Izaguirre M. A., Sander R., Smoydzyn L. (2008). Geophys. Res. Lett..

[cit22] Anastasio C., Newberg J. T. (2007). J. Geophys. Res..

[cit23] Dugourd P., Hudgins R. R., Jarrold M. F. (1997). Chem. Phys. Lett..

[cit24] Bradshaw J. A., Gordon S. L., Leavitt A. J., Whetten R. L. (2012). J. Phys. Chem. A.

[cit25] Eugene A.
J., Xia S.-S., Guzman M. I. (2016). J. Phys. Chem. A.

[cit26] Back R. A., Yamamoto S. (1985). Can. J. Chem..

[cit27] Lim Y. B., Kim H., Kim J. Y., Turpin B. J. (2016). Atmos. Chem. Phys..

[cit28] Kawamura K., Tachibana E., Okuzawa K., Aggarwal S. G., Kanaya Y., Wang Z. F. (2013). Atmos. Chem. Phys..

[cit29] Fu P., Kawamura K., Usukura K., Miura K. (2013). Mar. Chem..

[cit30] Bikkina S., Kawamura K., Miyazaki Y., Fu P. (2014). Geophys. Res. Lett..

[cit31] Herrmann H., Schaefer T., Tilgner A., Styler S. A., Weller C., Teich M., Otto T. (2015). Chem. Rev..

[cit32] Bock C. W., Redington R. L. (1988). J. Phys. Chem..

[cit33] Lin C.-L., Chu S.-Y. (1999). J. Am. Chem. Soc..

[cit34] Plath K. L., Axson J. L., Nelson G. C., Takahashi K., Skodje R. T., Vaidaa V. (2009). React. Kinet. Catal. Lett..

[cit35] Takahashi K., Plath K. L., Axson J. L., Nelson G. C., Skodje R. T., Vaida V. (2010). J. Chem. Phys..

[cit36] Olbert-Majkut A., Lundell J., Wierzejewska M. (2014). J. Phys. Chem. A.

[cit37] Mellouki A., Mu Y. (2003). J. Photochem. Photobiol., A.

[cit38] Reed Harris A. E., Ervens B., Shoemaker R. K., Kroll J. A., Rapf R. J., Griffith E. C., Monod A., Vaida V. (2014). J. Phys. Chem. A.

[cit39] Griffith E. C., Carpenter B. K., Shoemaker R. K., Vaida V. (2013). Proc. Natl. Acad. Sci. U. S. A..

[cit40] Carraway E. R., Hoffman A. J., Hoffmann M. R. (1994). Environ. Sci. Technol..

[cit41] Ekström G. N., McQuillan A. J. (1999). J. Phys. Chem. B.

[cit42] Ho C.-H., Shieh C.-Y., Tseng C.-L., Lin J.-L. (2008). J. Phys. Chem. C.

[cit43] Herburger A., van der Linde C., Beyer M. K. (2017). Phys. Chem. Chem. Phys..

[cit44] Dunbar R. C. (2004). Mass Spectrom. Rev..

[cit45] D. J. Wales, J. P. K. Doye, A. Dullweber, M. P. Hodges, F. Y. Naumkin F. Calvo, J. Hernández-Rojas and T. F. Middleton, The Cambridge Cluster Database, available at: http://www-wales.ch.cam.ac.uk/CCD.html, accessed 6 June 2017.

[cit46] Grimme S. (2006). J. Comput. Chem..

[cit47] Martin R. L. (2003). J. Chem. Phys..

[cit48] Lee S. Y., Brown R. C., Heller E. J. (1983). J. Phys. Chem..

[cit49] Ončák M., Šištík L., Slavíček P. (2010). J. Chem. Phys..

[cit50] Fuentealba P., Preuss H., Stoll H., von Szentpály L. (1982). Chem. Phys. Lett..

[cit51] Bergner A., Dolg M., Küchle W., Stoll H., Preuß H. (1993). Mol. Phys..

[cit52] Frisch M. J., Trucks G. W., Schlegel H. B., Scuseria G. E., Robb M. A., Cheeseman J. R., Scalmani G., Barone V., Petersson G. A., Nakatsuji H., Li X., Caricato M., Marenich A., Bloino J., Janesko B. G., Gomperts R., Mennucci B., Hratchian H. P., Ortiz J. V., Izmaylov A. F., Sonnenberg J. L., Williams-Young D., Ding F., Lipparini F., Egidi F., Goings J., Peng B., Petrone A., Henderson T., Ranasinghe D., Zakrzewski V. G., Gao J., Rega N., Zheng G., Liang W., Hada M., Ehara M., Toyota K., Fukuda R., Hasegawa J., Ishida M., Nakajima T., Honda Y., Kitao O., Nakai H., Vreven T., Throssell K., Montgomery Jr J. A., Peralta J. E., Ogliaro F., Bearpark M., Heyd J. J., Brothers E., Kudin K. N., Staroverov V. N., Keith T., Kobayashi R., Normand J., Raghavachari K., Rendell A., Burant J. C., Iyengar S. S., Tomasi J., Cossi M., Millam J. M., Klene M., Adamo C., Cammi R., Ochterski J. W., Martin R. L., Morokuma K., Farkas O., Foresman J. B. and Fox D. J., Gaussian 09, Revision A.02, 2016.

[cit53] Werner H.-J., Knowles P. J., Knizia G., Manby F. R., Schütz M., Celani P., Korona T., Lindh R., Mitrushenkov A., Rauhut G., Shamasundar K. R., Adler T. B., Amos R. D., Bernhardsson A., Berning A., Cooper D. L., Deegan M. J. O., Dobbyn A. J., Eckert F., Goll E., Hampel C., Hesselmann A., Hetzer G., Hrenar T., Jansen G., Köppl C., Liu Y., Lloyd A. W., Mata R. A., May A. J., McNicholas S. J., Meyer W., Mura M. E., Nicklass A., O‘Neill D. P., Palmieri P., Peng D., Pflüger K., Pitzer R., Reiher M., Shiozaki T., Stoll H., Stone A. J., Tarroni R., Thorsteinsson T. and Wang M., MOLPRO, version 2012.1, a package of ab initio programs, 2012.

[cit54] Prakash M. K., Weibel J. D., Marcus R. A. (2005). J. Geophys. Res..

[cit55] Domcke W., Yarkony D. R. (2012). Annu. Rev. Phys. Chem..

[cit56] Domcke W., Köppel H., Cederbaum L. S. (2006). Mol. Phys..

[cit57] Köppel H., Cederbaum L. S., Domcke W. (1982). J. Chem. Phys..

[cit58] Lesclaux R., Roussel P., Veyret B., Pouchan C. (1986). J. Am. Chem. Soc..

[cit59] Schröder D., Schalley C. A., Harvey J. N., Schwarz H. (1999). Int. J. Mass Spectrom..

[cit60] Weber J. M. (2014). Int. Rev. Phys. Chem..

[cit61] Compton R. N., Reinhardt P. W., Cooper C. D. (1975). J. Chem. Phys..

[cit62] Knapp M., Echt O., Kreisle D., Märk T. D., Recknagel E. (1986). Chem. Phys. Lett..

[cit63] Janik I., Tripathi G. N. R. (2016). J. Chem. Phys..

[cit64] Denifl S., Vizcaino V., Märk T. D., Illenberger E., Scheier P. (2010). Phys. Chem. Chem. Phys..

[cit65] Balaj O. P., Siu C.-K., Balteanu I., Beyer M. K., Bondybey V. E. (2004). Chem. – Eur. J..

[cit66] Arnold S. T., Morris R. A., Viggiano A. A., Johnson M. A. (1996). J. Phys. Chem..

[cit67] Akhgarnusch A., Tang W. K., Zhang H., Siu C.-K., Beyer M. K. (2016). Phys. Chem. Chem. Phys..

[cit68] Lengyel J., van der Linde C., Akhgarnusch A., Beyer M. K. (2017). Int. J. Mass Spectrom..

[cit69] Tsukuda T., Nagata T. (2003). J. Phys. Chem. A.

[cit70] Höckendorf R. F., Balaj O. P., van der Linde C., Beyer M. K. (2010). Phys. Chem. Chem. Phys..

[cit71] Zhou M., Andrews L. (1999). J. Chem. Phys..

[cit72] Thompson W. E., Jacox M. E. (1999). J. Chem. Phys..

[cit73] Ovenall D. W., Whiffen D. H. (1961). Mol. Phys..

[cit74] Hartman K. O., Hisatsune I. C. (1966). J. Chem. Phys..

[cit75] Vayenas C. G., White R. E. and Gamboa-Aldeco M., Modern Aspects of Electrochemistry, Springer Science + Business Media, New York, 2008, vol. 42.

[cit76] Gueymard C. A., Myers D., Emery K. (2002). Sol. Energy.

[cit77] National Renewable Energy Laboratory (NREL), Reference Solar Spectral Irradiance: Air Mass 1.5, available at: http://rredc.nrel.gov/solar/spectra/am1.5/.

