# Supplementary material for: Photochemistry of glyoxylate embedded in sodium chloride clusters, a laboratory model for tropospheric sea-salt aerosols
Source: Phys Chem Chem Phys. 2018 Feb 28;20(12):8143–51. doi: 10.1039/c8cp00399h (PMC5885371; doi:10.1039/c8cp00399h)
Supplement: Supplementary file 1 [file CP-020-C8CP00399H-s001.pdf]

Electronic Supplementary Material (ESI) for Physical Chemistry Chemical Physics.
This journal is © the Owner Societies 2018

Photochemistry of glyoxylate embedded in sodium chloride clusters, a laboratory model for tropospheric sea-salt aerosols

Nina K. Bersenkowitsch, Milan Ončák, Christian van der Linde, Andreas Herburger, Martin K. Beyer

Institut für Ionenphysik und Angewandte Physik, Universität Innsbruck, Technikerstraße 25, 6020 Innsbruck, Austria

Email: milan.oncak@uibk.ac.at; martin.beyer@uibk.ac.at

Electronic supplementary information (ESI)

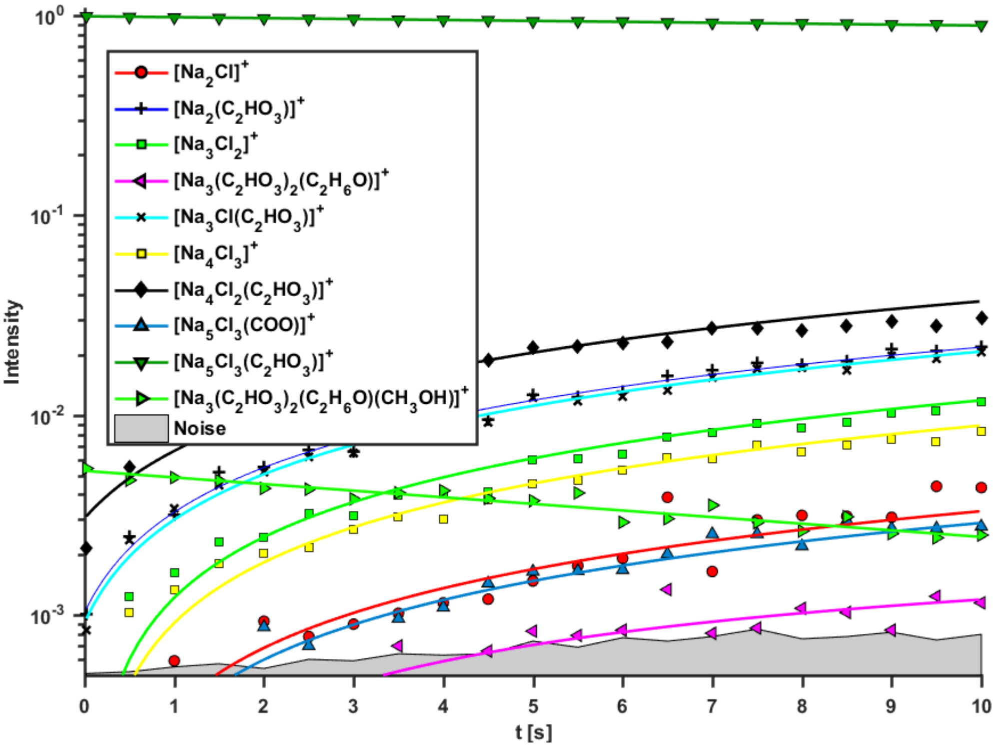

Figure S1: Kinetics of the cluster $[Na_5Cl_3(C_2HO_3)]^+$ at 335 nm and 20 accumulations. The linear decrease of the precursor ion with varying pulse numbers show that the fragmentation occurs due to single photon processes. Another peak, which was identified as $[Na_3(C_2HO_3)_2(C_2H_6O)(CH_3OH)]^+$, with very little intensity was directly situated beside the parent ion. Due to the very small mass difference of 0.17 m/z, it was not possible to eliminate this peak without exciting $[Na_5Cl_3(C_2HO_3)]^+$ in a way that enough signal remained. Therefore it contributes also to the first and fourth fragment listed in the legend. Due to the low intensity of this fragmenting cluster, the influence on the cross section is negligible.

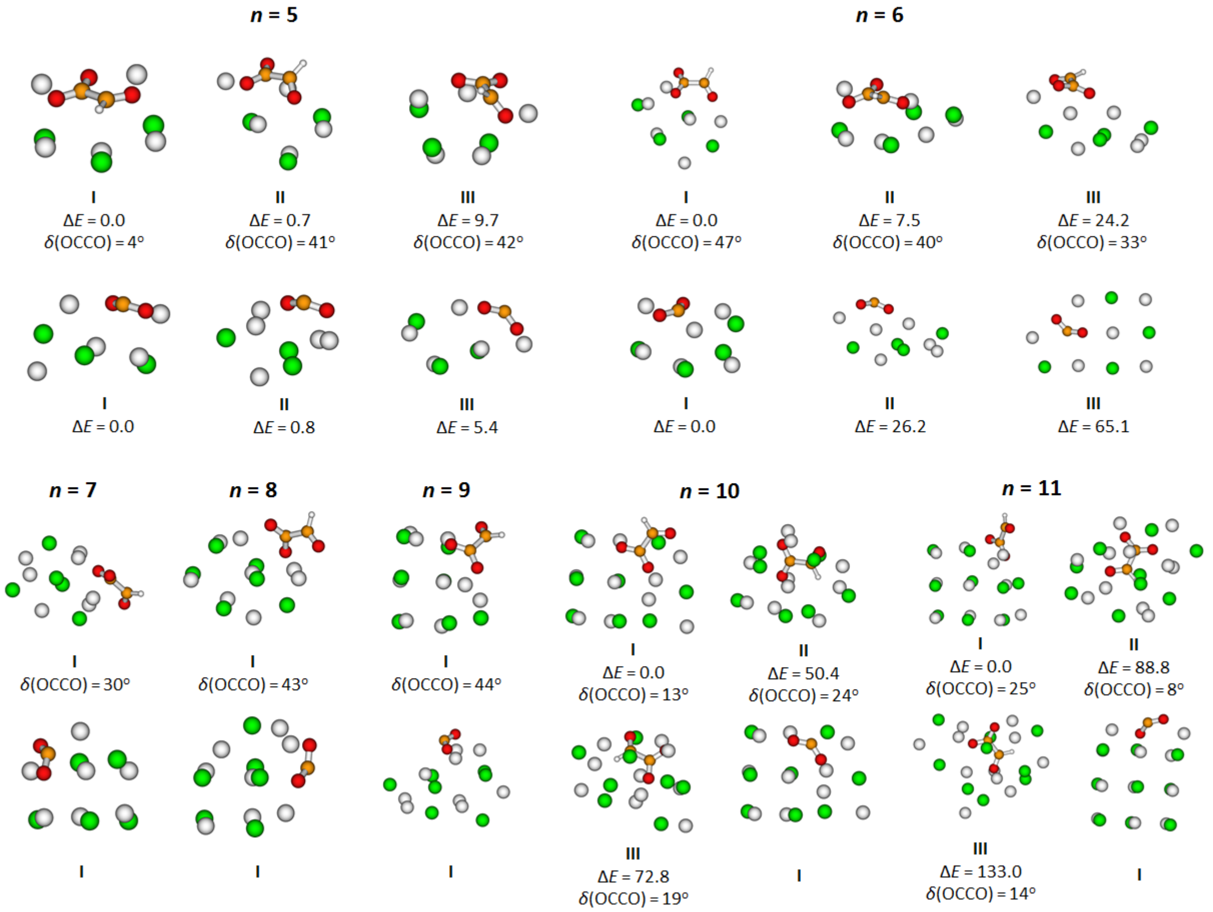

Figure 2: Structure, relative energy *ΔE* (in kJ mol^{-1}) and dihedral angles *δ* of the most stable $[Na_nCl_{n-2}C_2HO_3]^+$ and $[Na_nCl_{n-2}CO_2]^+$ isomers found, *n* = 5–11. For *n* = 10,11, isomers with $C_2HO_3^-$ inside the cluster are shown for comparison. Energy was calculated at the B3LYP+D2/def2TZVP//B3LYP+D2/6-31+g* level of theory. Zero point correction is included in all reported energies.

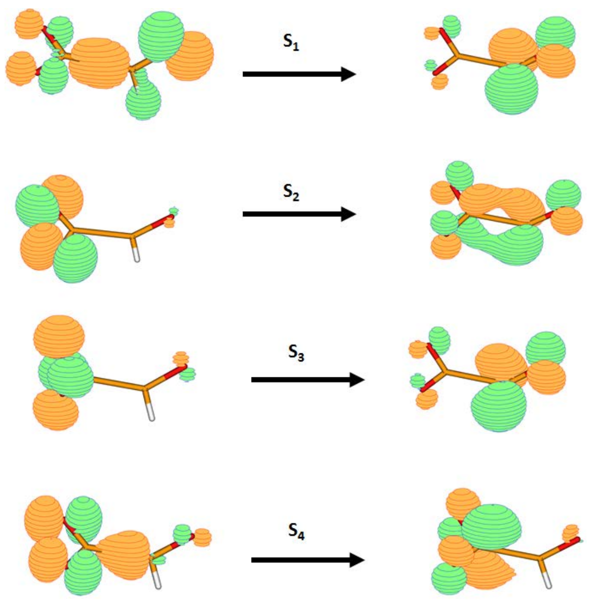

Figure S3: Natural transition orbitals (NTO) involved in the formation of excited states of the $C_2HO_3^-$ ion, calculated at the TD-BHandHLYP/def2TZVP level of theory.

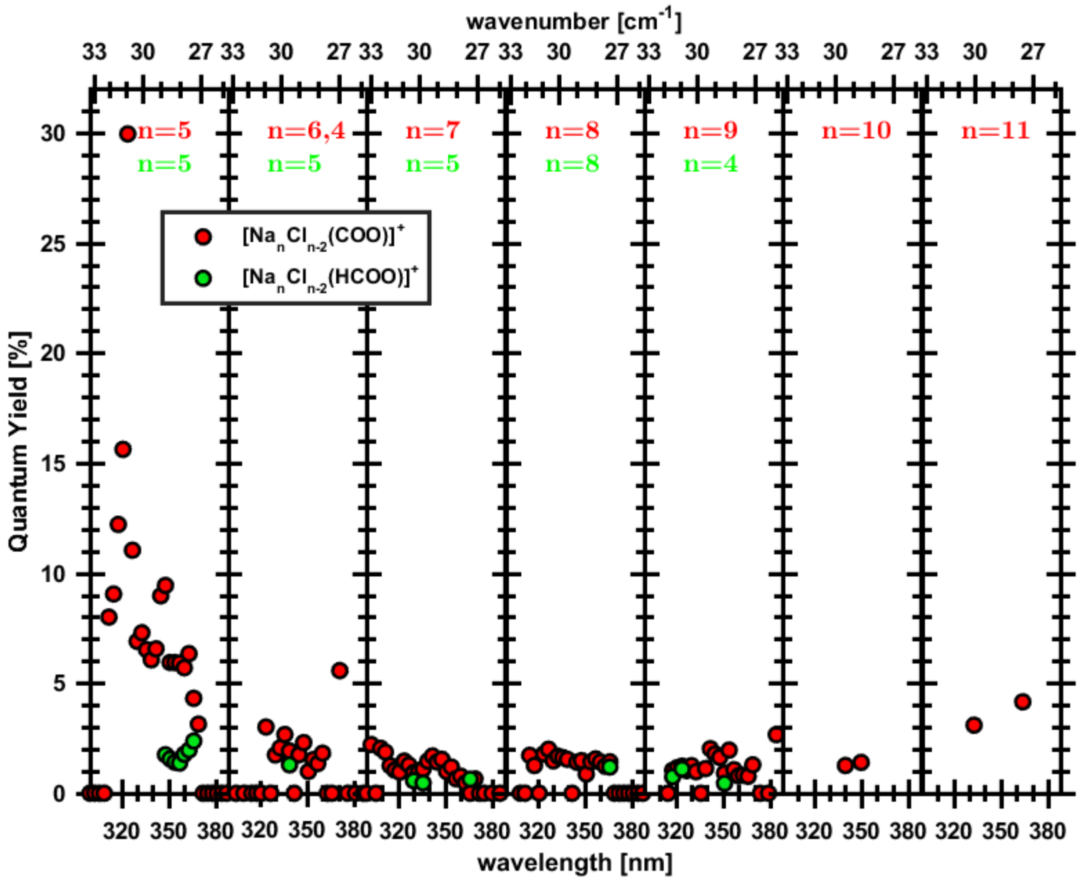

Figure S4: Quantum Yields for the fragments containing carbon dioxide anion radical (red line) and HCOO (green bullets).

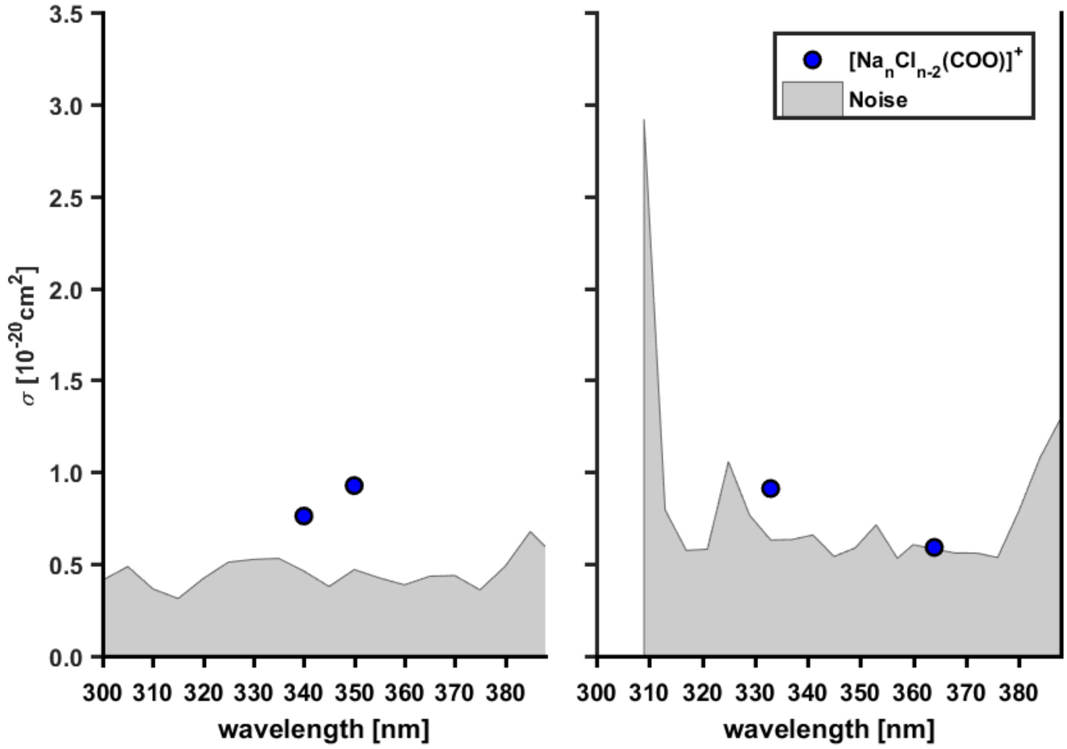

Figure S5: Quantum Yields for the fragments including carbon dioxide anion radical for the clusters $[Na_nCl_{n-2}(C_2HO_3)]^+$, n=10,11. The products are formed hardly above the noise level.

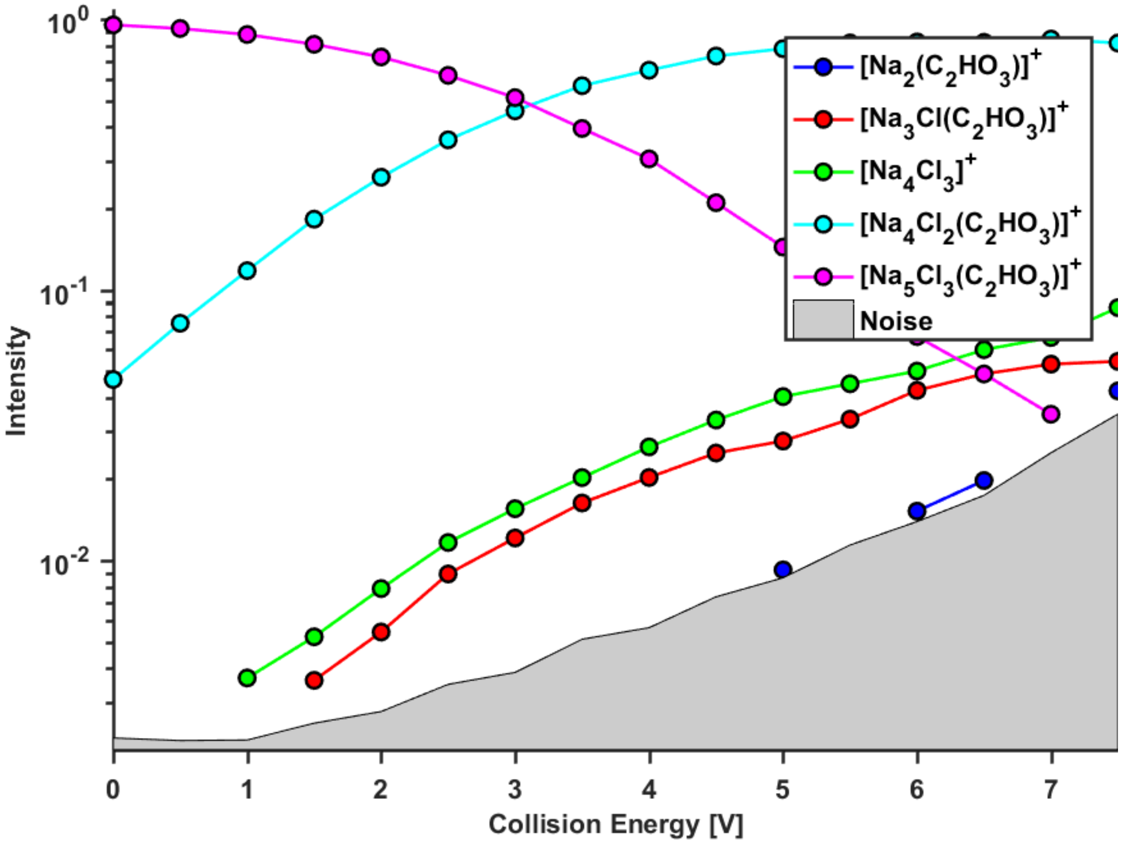

Figure S6: Collision induced dissociation of $Na_5Cl_3(C_2HO_3)^+$ in the hexapole collision cell. There is no evidence for C-C bond cleavage, since only stoichiometric fragments are observed.

Table S1: Relative energies Δ*E* (in kJ mol^{-1}) and dihedral angle *δ* (in degrees) of various isomers of $[Na_5Cl_3(C_2HO_3)]^+$ optimized at the B3LYP+D2/def2TZVP and B3LYP/def2TZVP levels of theory.

	B3LYP+D2/ def2TZVP		B3LYP/def2TZVP	
isomer	Δ*E*	*δ*	Δ*E*	*δ*
**I**	0.0	6	12.4	14
**II**	1.1	45	16.7	89
**III**	10.1	44	12.4	14
**IV**	16.9	45	27.1	46
**V**	21.1	42	27.2	43
**VI**	23.3	6	0.0	4

Benchmarking of excited state calculations

Table S2 includes benchmarking of excitation energies of $C_2HO_3^-$, $[Na_2(C_2HO_3)]^+$, $[Na_3Cl(C_2HO_3)]^+$ and $[Na_5Cl_3(C_2HO_3)]^+$ ions. It can be seen that for $C_2HO_3^-$, results of the TD-BHandHLYP method are in much better agreement with the EOM-CCSD results than the ones of TD-CAMB3LYP. Oscillator strengths are comparable among all used methods, with the exception of S_2 and S_6 state whose oscillator strengths are underestimated at the TD-BHandHLYP level. There are only minor basis set effects.

For larger clusters, both DFT functionals predict low-lying states of charge-transfer character (the transfer takes place between Cl^- and $C_2HO_3^-$, i.e. forming doubly-charged $C_2HO_3^{2-}$). These states are not reproduced at the EOM-CCSD level and we therefore conclude that they are artificial.

Table S2: Excitation energies *E* (in eV) and oscillator strengths *f* of several investigated ions as calculated using various approaches in the structure optimized at the B3LYP+D2/def2TZVP level of theory.

method		EOMCCSD/def2TZVP		EOMCCSD/aug-cc-pVDZ		EOMCCSD/cc-pVDZ		TD-BHandHLYP/def2TZVP		TD-CAMB3LYP/def2TZVP	
ion	State	*E*	*f*	*E*	*f*	*E*	*f*	*E*	*f*	*E*	*f*
$C_2HO_3^-$	S_1	3.88	0.0011	-	-	3.85	0.0013	3.91	0.0009	3.67	0.0009
	S_2	5.66	0.0464	-	-	5.65	0.0472	5.73	0.0319	5.23	0.0422
	S_3	5.98	0.0120	-	-	6.02	0.0113	5.87	0.0110	5.39	0.0085
	S_4	6.14	0.0118	-	-	6.18	0.0121	6.12	0.0100	5.82	0.0099
	S_5	6.71	0.0128	-	-	6.82	0.0144	6.74	0.0086	6.28	0.0082
	S_6	7.00	0.0486	-	-	7.06	0.0422	7.15	0.0355	6.66	0.0217
$[Na_2(C_2HO_3)]^+$	S_1	3.81	0.0002	3.81	0.0004	3.83	0.0002	3.83	0.0002	3.61	0.0002
	S_2	5.31	0.0000	5.28	0.0000	5.30	0.0000	5.31	0.0000	4.83	0.0000
	S_3	6.20	0.0872	6.13	0.0845	6.17	0.0879	6.15	0.0645	5.71	0.0502
	S_4	6.22	0.0000	6.21	0.0001	6.26	0.0001	6.25	0.0000	5.87	0.0000
	S_5	7.10	0.0376	6.88	0.0364	6.79	0.0397	6.90	0.0384	6.44	0.0338
	S_6	7.35	0.0032	7.15	0.0036	7.02	0.0032	7.08	0.0013	6.69	0.0201
$[Na_3Cl(C_2HO_3)]^+$	S_1	3.80	0.0001	3.79	0.0002	3.82	0.0001	3.80	0.0001	3.58	0.0001
	S_2	5.43	0.0000	5.40	0.0000	5.45	0.0001	4.81	0.0025	4.63	0.0029
	S_3	6.09	0.0004	6.08	0.0008	6.13	0.0367	5.07	0.0004	4.82	0.0003
	S_4	6.32	0.0924	6.24	0.0703	6.15	0.0370	5.20	0.0000	5.00	0.0001
	S_5	6.37	0.0718	6.26	0.0898	6.33	0.0963	5.47	0.0000	5.02	0.0000
	S_6	6.54	0.0013	6.47	0.0010	6.47	0.0496	5.78	0.0338	5.58	0.0413
$[Na_5Cl_3(C_2HO_3)]^+$	S_1	-	-	3.88	0.0006	3.91	0.0003	3.92	0.0005	3.68	0.0006
	S_2	-	-	5.25	0.0003	5.28	0.0004	5.29	0.0019	4.79	0.0010
	S_3	-	-	6.00	0.0179	6.05	0.0164	5.42	0.0347	5.03	0.0310
	S_4	-	-	6.12	0.0226	6.17	0.0258	5.52	0.0314	5.13	0.0340

Cartesian coordinates and energy (in Ångstrom and Hartree, respectively) of structures included in the manuscript

Structures optimized at the B3LYP+D2/def2TZVP level of theory

C2HO3-
E=-302.639808
C -0.595211 0.022156 0.027260
O -1.150479 -1.083327 -0.170202
O -1.043545 1.180884 0.021645
C 0.885883 -0.183715 0.367777
H 1.044748 -0.742778 1.326933
O 1.845426 0.116459 -0.313587

CHO-
E=-113.896773
C 0.079244 0.628114 0.000000
H -1.109415 1.050824 0.000000
O 0.079244 -0.602438 0.000000

CHO
E=-113.891303
C 0.061785 0.582494 0.000000
H -0.864996 1.220692 0.000000
O 0.061785 -0.589457 0.000000

CO2-
E=-188.636373
C 0.000000 0.341429 0.000000
O 1.144167 -0.128494 0.000000
O -1.144167 -0.127578 0.000000

CO2
E=-188.658348
C 0.000000 0.000000 -0.000052
O 0.000000 0.000000 -1.159716
O 0.000000 0.000000 1.159755

CO
E=-113.357481
C 0.000000 0.000000 -0.642728
O 0.000000 0.000000 0.482046

NaCl
E=-622.604860
Cl 0.000000 0.000000 0.932933
Na 0.000000 0.000000 -1.441805

Na2C2HO3+
E=-627.134431
C -0.987514 1.338271 0.000022
O -2.078647 0.817372 0.000046
C 0.319620 0.515759 0.000005
O 0.226401 -0.752148 -0.000010
O 1.370854 1.172224 0.000019
H -0.859477 2.431036 0.000095
Na -1.899561 -1.470479 -0.000021
Na 2.692104 -0.661775 -0.000043

Na2Cl2CO
E=-1358.653922

Na -0.653577 -0.002950 -0.002225
Cl 0.928423 -2.037954 -0.000217
Na 2.470220 0.004595 0.001787
Cl 0.918493 2.039695 -0.000217
O -3.186752 -0.003875 0.000391
C -4.314437 -0.002781 0.001515

Na2Cl2
E=-1245.290151
Cl -2.034618 -0.000004 -0.000006
Na 0.000008 -1.560793 -0.000007
Na -0.000009 1.560792 -0.000007
Cl 2.034619 0.000004 0.000015

Na2ClC2HO3
E=-1087.635966
C -2.470320 -0.762036 0.000167
C -1.806774 0.637570 0.000265
O -2.573930 1.589306 -0.000444
O -1.852719 -1.804793 0.000157
O -0.533026 0.622492 0.000563
Na 1.316926 1.705858 -0.000500
Na 0.504463 -1.375646 0.000492
H -3.574089 -0.748572 -0.000172
Cl 2.875222 -0.317236 -0.000267

Na2ClHCOO
E= -974.277987
C 2.284248 0.361080 0.000280
O 1.178593 0.984400 0.000041
O 2.414193 -0.875524 -0.000278
Na -0.832649 1.651022 -0.000204
Na 0.178663 -1.322787 -0.000041
Cl -2.262012 -0.448814 0.000107
H 3.200271 0.981753 0.001089

Na2Cl+
E=-784.774210
Na 0.000000 0.000000 2.497394
Cl 0.000000 0.000000 0.000000
Na 0.000000 0.000000 -2.497394

Na3Cl2C2HO3
E=-1710.317310
C 2.346027 -2.036377 0.000640
O 3.171170 -1.154014 0.001351
C 0.820708 -1.783551 -0.000080
O 0.420519 -0.587398 0.000379
O 0.131066 -2.817503 -0.001155
Na -1.917695 -1.836348 -0.001228
Na -1.084167 1.414147 0.000377
Na 2.193513 0.977846 -0.000335
Cl 0.870489 3.112324 -0.000611
H 2.634905 -3.100485 0.000821
Cl -3.371990 0.204104 0.000862

Na3Cl2+
E=-1407.437993
Na 0.000000 0.000000 0.000000
Cl 0.000000 0.000000 2.571363
Na 0.000000 0.000000 -5.032304
Cl 0.000000 0.000000 -2.571363

Na 0.000000 0.000000 5.032304

Na3Cl3
E=-1867.958624
Cl -0.000004 1.833228 0.000225
Na -2.512589 1.170623 0.000023
Na 2.512581 1.170627 0.000063
Cl 2.604369 -1.331204 -0.000042
Cl -2.604361 -1.331211 -0.000005
Na 0.000002 -1.059778 -0.000359

Na3ClC2HO3+
E=-1249.802884
C 1.004786 0.405273 0.000320
O -0.119852 0.932486 0.000235
O 1.258839 -0.831170 0.000238
C 2.210412 1.375649 0.000255
O 3.351891 0.981929 0.000125
H 1.964609 2.448969 0.000118
Na 3.456173 -1.329973 0.000226
Na -1.076912 -1.454061 -0.000213
Cl -3.425501 -0.562828 -0.000624
Na -2.283744 1.671998 0.000191

Na3Cl2COOH
E= -1596.953677
C -0.215288 -1.951129 0.000479
O -0.649659 -1.554980 -1.116128
O -0.649824 -1.554333 1.116857
Na 0.563462 0.401087 1.549328
Na 0.561265 0.400169 -1.549691
Cl -1.146766 1.905161 -0.000025
Na -2.360026 -0.369253 0.000315
H 0.602458 -2.689497 0.000753
Cl 2.598144 0.125353 -0.000500

Na3ClCOOH+
E=-1136.445487
C 1.848649 0.000047 -0.463519
O 1.447858 -1.115708 -0.037732
O 1.447792 1.115784 -0.037756
Na -0.420595 2.069783 -0.771861
Cl -1.826551 -0.000054 0.126210
Na -0.420474 -2.069802 -0.771865
Na 0.312503 0.000016 1.771243
H 2.608501 0.000058 -1.263246

Na4Cl2COOH+
E=-1759.133531
H -0.004882 -3.666818 -0.659599
C -0.003493 -2.666020 -0.197488
O 1.107909 -2.135754 0.050154
O -1.113434 -2.133268 0.051434
Na 2.923618 -1.049734 -0.391431
Na -0.000209 -0.187143 1.016560
Na -2.926444 -1.042925 -0.390726
Cl 2.035494 1.373897 0.119504
Na 0.003769 2.918064 -0.508510
Cl -2.031849 1.378704 0.118555

Na4Cl3+
E=-2030.139930

Na 0.000000 -2.658045 -0.703223
Cl 0.000000 2.311568 0.123147
Na -2.301934 1.329022 -0.703223
Cl 2.001876 -1.155784 0.123147
Na 2.301934 1.329022 -0.703223
Cl -2.001876 -1.155784 0.123147
Na 0.000000 0.000000 1.538714

Na5Cl3COH+
E=-2306.436405
Na 1.804949 -0.001219 1.696637
Cl 1.835433 1.984273 -0.083299
Na -3.566208 0.000502 0.007837
Na -0.694557 1.805329 0.180652
Cl -1.460737 0.001545 -1.695416
Na 1.435289 0.000650 -1.849042
Na -0.695690 -1.805319 0.176810
Cl 1.834400 -1.984664 -0.084581
C -1.891157 -0.001309 1.971932
H -2.159977 -0.003228 3.059663
O -0.646166 -0.000989 1.805369

Na5Cl3+
E=-2192.449317
Na -3.076154 -1.721797 -0.427679
Cl -0.585654 -2.425862 0.289344
Na 1.883027 -2.445352 -0.603496
Cl 2.616732 0.000181 -0.003838
Na 0.153585 -0.000012 1.173902
Cl -0.586022 2.425713 0.289275
Na 1.882757 2.445679 -0.603325
Na -3.076482 1.721431 -0.427700

Na5Cl3COOH+
E=-2381.812018
Na -0.846472 -1.346430 0.845614
Na 1.819165 -1.208998 -1.279435
Cl 1.632542 -1.760890 1.327168
Na 1.730318 0.904755 1.583265
Cl 2.384464 1.381075 -0.973663
Cl -1.111204 1.379261 1.275747
Na -3.542378 0.568233 0.514312
Na -0.144379 1.508004 -1.274497
C -1.802211 -1.018771 -1.718650
O -2.634627 -0.971859 -0.777963
H -2.181850 -1.254439 -2.726787
O -0.563162 -0.816230 -1.589590

NaC2H3O
E=-464.951983
C 0.369266 1.164589 0.000166
O -0.820280 1.428360 -0.000010
C 0.960577 -0.276495 -0.000135
O 0.115241 -1.220343 0.000303
O 2.186935 -0.298781 -0.000165
Na -1.905116 -0.597713 -0.000142
H 1.122041 1.972388 0.000359

NaCOOH
E=-351.596854
C 1.052620 -0.000504 -0.000021
O 0.476876 1.120404 0.000040

O 0.475323 -1.120515 0.000040
H 2.160254 -0.001263 -0.000257
Na -1.463052 0.000471 -0.000024

NaClCO
E=-735.969427
Cl -2.379654 0.354644 0.000000
Na 0.000000 0.225456 0.000000
C 3.500455 -0.814776 0.000000
O 2.431424 -0.452539 0.000000

Na5Cl3C2HO3+, isomer I
E=-2495.184208
na -2.629105 1.683740 -0.776296
cl -2.547822 -0.869536 -1.330798
na -2.197781 -1.149735 1.320902
cl 0.357747 -1.916159 1.260453
na 0.031175 -1.583521 -1.461245
cl 2.397707 -0.301025 -1.720834
na 2.790581 -0.956108 0.812161
na 1.758607 2.091800 -0.878452
c -0.566334 1.469970 0.967856
o -0.364449 2.090168 -0.106092
o -1.674493 1.114468 1.420522
c 0.668471 1.074758 1.808538
o 1.803445 1.248034 1.412123
h 0.453149 0.636554 2.791497

Na5Cl3C2HO3+, isomer II
E=-2495.183787
C 0.785631 2.150014 0.876035
O -0.288684 2.168259 0.313626
C 1.933401 1.313084 0.306914
O 2.481981 0.550759 1.139416
O 2.146354 1.371499 -0.922213
Na 0.021561 0.735810 -1.779440
Na 1.048422 -1.127427 1.865899
Na -2.548567 1.366996 0.671160
Na 3.380971 -0.630043 -0.874092
Cl -2.560843 0.215013 -1.702707
Cl -1.522358 -0.687973 2.051053
Na -1.783559 -1.930998 -0.352630
Cl 0.948795 -1.803986 -0.820667
H 0.946371 2.647622 1.845277

Na5Cl3C2HO3+, isomer III
E=-2495.180377
C -1.381382 0.949179 -1.339501
O -1.920012 1.765902 -0.560258
C -2.122844 -0.346649 -1.668391
O -2.668223 -0.986859 -0.784952
H -2.049191 -0.735646 -2.695474
Na 1.855104 0.022175 -1.855178
Na -1.013478 -2.287406 0.489851
Na -3.028947 0.686061 1.059625
Na 0.296057 2.186613 0.393379
Cl 2.791199 1.430420 0.214341
Na 2.071535 -0.729814 1.515783
Cl 1.334352 -2.301036 -0.558599
Cl -0.620325 -0.046885 2.006134
O -0.223925 0.978964 -1.798185

Na5Cl3C2HO3+, isomer IV
E=-2495.177778
C -3.488551 1.029351 -1.020994
C -2.297836 0.090153 -1.243044
O -2.468150 -1.120887 -0.980676
O -1.223333 0.653801 -1.560664
O -4.143194 0.951500 -0.004699
H -3.671594 1.816844 -1.770457
Na -3.272535 -0.910591 1.218808
Cl -0.643931 -0.275559 1.695048
Na 2.070234 -0.396151 1.751415
Na -0.153161 -1.536896 -0.819776
Na -0.022100 1.824616 -0.019670
Cl 2.586077 1.889005 0.375000
Na 3.758935 0.265192 -1.313040
Cl 2.462103 -1.855626 -0.497819

Na5Cl3C2HO3+, isomer V
E=-2495.176177
C -2.786678 1.380307 -1.195351
C -1.709602 0.284950 -1.196684
O -0.525019 0.705279 -1.083279
O -3.768368 1.273931 -0.497643
O -2.093496 -0.896409 -1.207123
Na 1.675447 0.728102 -1.701543
Na -0.169056 -1.823517 -0.194093
Na -3.618124 -0.817450 0.656097
Cl 2.382105 -1.731594 -0.897464
Na 3.825800 -0.432133 0.864026
Cl -1.323820 -0.730763 2.026853
H -2.595455 2.286365 -1.794261
Cl 2.551604 1.848656 0.587331
Na 0.040487 1.389780 1.217931

Na5Cl3C2HO3+, isomer VI
E=-2495.175326
c 2.796060 0.605381 -0.300566
o 1.745173 -0.055051 -0.103403
o 2.941915 1.838799 -0.336360
c 4.101240 -0.201622 -0.513994
o 4.154233 -1.402053 -0.391783
h 4.989563 0.397398 -0.770844
na -0.564049 -0.040110 0.184743
cl -0.433964 -2.755367 0.758218
na 2.074991 -2.328361 0.169234
na 1.385833 3.278156 0.170522
cl -1.072248 2.646907 0.673077
na -3.468496 2.123916 -0.190589
cl -3.162208 -0.360060 -0.838353
na -2.859261 -2.843479 -0.131133

Na5Cl3CO2+, isomer I
E=-2381.165358
Na -3.098281 -1.232454 -0.587678
Cl -2.108035 0.940324 -1.623255
Na -1.699119 1.763567 0.902581
Cl 1.006481 1.385267 1.017385
Na 3.391340 1.317351 -0.210203
Cl 2.807753 -1.106252 -0.948417
Na 1.435164 -1.398382 1.324620
Na 0.278120 -0.194863 -1.259154
C -1.254919 -1.062842 1.408379

O -0.793952 -1.679133 0.424453
O -2.312964 -0.465757 1.588144

Na5Cl3CO2+, isomer II
E=-2381.164996
Cl 2.341221 1.728724 0.177688
Na -0.169633 2.083893 0.517688
Na 1.914492 -0.466898 1.623631
Na 1.626569 0.103106 -1.826661
Cl 1.658732 -2.168071 -0.407865
Cl -1.197391 0.565706 -1.586472
Na -0.859123 -1.821530 -0.079363
Na -3.645674 0.363453 -0.508673
C -1.724814 0.090167 1.651566
O -0.502043 -0.028327 1.781397
O -2.601412 -0.668094 1.216201

Na5Cl3CO2+, isomer III
E=-2381.163338
Na -3.293422 0.538089 -1.110594
Cl -2.361261 -1.719548 -0.156731
Cl -1.732604 2.067170 0.338457
Na -1.472557 -0.154040 1.859734
Na 0.200406 -1.888929 -0.786676
Cl 1.208543 -0.559747 1.484298
Na 0.776647 1.592275 -0.351283
Na 3.790910 -0.110488 0.872249
C 2.445755 -0.102026 -1.638384
O 3.034076 0.802629 -1.023019
O 1.260191 -0.243593 -1.953211

Na5Cl3CO2+, isomer IV
E=-2381.162867
Na -4.020715 -0.118440 -0.163417
Cl -1.928696 -0.931146 -1.531772
Na -0.805591 1.422830 -0.690541
Na -0.216311 -1.929785 0.278451
Na 1.652841 0.596894 1.711792
Cl 2.400425 -1.692039 0.498702
Na 3.182560 -0.074346 -1.411534
Cl 1.778103 1.988335 -0.596563
O -0.585001 0.121614 1.532054
C -1.802630 0.338306 1.655871
O -2.558997 1.115126 1.067477

Na5Cl3CO2+, isomer V
E=-2381.157699
Na -1.633939 -3.008609 -0.001013
Cl 0.936343 -2.701852 0.000940
Na 3.396136 -1.849381 0.000547
Cl 2.945757 0.710500 -0.001048
Na 0.259942 -0.013411 0.000227
Cl -0.504794 2.636060 0.000882
Na 2.050137 3.148969 -0.000220
Na -2.963937 1.809826 -0.000240
C -3.267871 -0.881667 -0.000145
O -2.025844 -0.694049 -0.001102
O -4.223993 -0.134869 0.000525

Structures optimized at the B3LYP+D2/6-31+g* level of theory

CHO
E=-113.845662
C 0.062303 0.589088 0.000000
H -0.872241 1.216161 0.000000
O 0.062303 -0.593836 0.000000

[Na5Cl3C2HO3]+, isomer I
E=-2494.926396
na 2.596868 1.727683 0.769220
cl 2.600191 -0.841068 1.318828
na 2.249578 -1.117445 -1.342055
cl -0.321365 -1.873673 -1.240742
na 0.007385 -1.530926 1.490592
cl -2.416361 -0.338880 1.715331
na -2.805345 -0.986358 -0.829133
na -1.812249 2.067352 0.857444
c 0.536071 1.426076 -0.954876
o 0.320503 2.007962 0.147510
o 1.662074 1.122648 -1.423426
c -0.691995 1.036622 -1.808472
o -1.838492 1.213111 -1.412960
h -0.477672 0.622218 -2.803726

[Na5Cl3C2HO3]+, isomer II
E=-2494.925716
C 0.713049 2.208671 0.648405
O -0.350216 2.192227 0.046843
C 1.885038 1.351276 0.155194
O 2.479662 0.695848 1.055783
O 2.082621 1.286347 -1.084585
Na -0.012711 0.545898 -1.861577
Na 1.132536 -0.929585 1.981940
Na -2.577616 1.428580 0.544815
Na 3.387617 -0.643387 -0.850259
Cl -2.592102 -0.023218 -1.670332
Cl -1.443593 -0.450599 2.109518
Na -1.752534 -2.012192 -0.113446
Cl 0.972584 -1.867172 -0.632030
H 0.837616 2.759299 1.596204

[Na5Cl3C2HO3]+, isomer III
E=-2494.921786
C 1.331676 -0.987948 -1.338964
O 1.878414 -1.807845 -0.555596
C 2.067653 0.313392 -1.669771
O 2.655896 0.934618 -0.787501
H 1.957575 0.731671 -2.684035
Na -1.884012 -0.035484 -1.862578
Na 1.092841 2.315049 0.473963
Na 3.029915 -0.734911 1.030200
Na -0.321777 -2.191579 0.432223
Cl -2.814600 -1.375998 0.267029
Na -2.048514 0.801598 1.533166
Cl -1.261466 2.291930 -0.599310
Cl 0.635409 0.071084 1.987055
O 0.163770 -1.022400 -1.790832

[Na5Cl3CO2]+, isomer I
E=-2380.948527

Na 3.113889 -1.266858 0.531452
Cl 2.112674 0.881523 1.651787
Na 1.707602 1.810833 -0.856600
Cl -1.005544 1.400467 -0.966830
Na -3.419919 1.327513 0.225088
Cl -2.822657 -1.128227 0.910288
Na -1.421373 -1.389587 -1.360514
Na -0.280213 -0.247005 1.269348
C 1.255531 -0.990048 -1.435820
O 0.792716 -1.649981 -0.469413
O 2.323652 -0.382212 -1.580682

[Na5Cl3CO2]+, isomer II
E=-2380.947859
Cl 2.358052 -1.708535 -0.178208
Na -0.156885 -2.091402 -0.527509
Na 1.907421 0.481585 -1.647600
Na 1.633495 -0.101878 1.854532
Cl 1.643995 2.168441 0.411973
Cl -1.193166 -0.580529 1.578183
Na -0.881126 1.821954 0.080319
Na -3.657569 -0.366904 0.507130
C -1.724109 -0.096099 -1.665641
O -0.488495 0.017762 -1.764386
O -2.599633 0.663525 -1.203720

[Na5Cl3CO2]+, isomer III
E=-2380.946955
Na -3.324741 0.547411 -1.115503
Cl -2.362538 -1.706560 -0.151139
Cl -1.727212 2.055153 0.336063
Na -1.473250 -0.156631 1.892795
Na 0.203466 -1.898306 -0.802459
Cl 1.205910 -0.559408 1.475710
Na 0.787994 1.596198 -0.375186
Na 3.795544 -0.094560 0.886746
C 2.457132 -0.113653 -1.644835
O 3.041124 0.795299 -1.013137
O 1.259295 -0.253983 -1.950875

[Na6Cl4C2HO3]+, isomer I
E=-3117.566080
Na 2.841097 1.003424 1.046286
Cl 3.155176 -1.582063 0.535180
Na 0.763059 -1.999769 1.564689
Cl 0.150014 0.649564 1.783952
Na -2.124300 2.109243 1.334616
Cl -3.585772 0.170903 0.191054
Na -3.281686 -2.355101 -0.391305
Cl -0.622090 -2.448182 -0.774588
Na 1.794079 -1.270473 -1.727140
C 1.236240 1.888095 -1.274800
Na -1.080693 0.270062 -0.775143
C 0.182097 2.988963 -1.102203
O 2.333186 2.203213 -0.771430
O 0.876650 0.792889 -1.793291
O -0.920476 2.747360 -0.622504
H 0.493406 4.024927 -1.317406

[Na6Cl4C2HO3]+, isomer II
E=-3117.563087
C -0.529978 2.882134 -0.733845

O 0.643403 2.714775 -0.443307
C -1.433783 1.703144 -1.119864
O -0.946388 0.796065 -1.843527
O -2.592125 1.735959 -0.625420
Na 2.054611 2.089503 1.381572
Na -2.768833 0.734821 1.428924
Na 1.093627 0.219807 -0.943189
Na -2.882039 -0.511505 -1.778834
Cl -0.067595 0.402458 1.746008
Cl 3.673022 0.629323 -0.071831
Na -0.608950 -2.248670 1.120410
Cl -3.167411 -1.736824 0.533864
H -1.006425 3.875545 -0.661995
Na 3.767468 -1.972753 -0.273244
Cl 1.252287 -2.517594 -0.749792

[Na6Cl4C2HO3]+, isomer III
E=-3117.556206
Na -1.570054 1.892514 -0.866802
C -2.131739 -0.673053 -1.541626
Na -4.331256 -0.293561 0.289543
Cl -2.586542 1.258239 1.589658
Na -0.073962 0.667922 2.150035
Cl 1.199869 -1.626651 1.600071
Na 3.706894 -2.055806 1.053146
Cl 3.956763 -0.469130 -1.039316
Na 3.581820 2.094807 -0.701639
Cl 1.041483 2.108897 0.012071
Na 1.187448 -0.554238 -0.929867
O -0.897605 -0.441002 -1.645482
O -3.057948 0.176871 -1.576405
C -2.512899 -2.128133 -1.216955
H -1.873569 -2.927339 -1.631739
O -3.440092 -2.379193 -0.467810

[Na6Cl4CO2]+, isomer I
E=-3003.593352
C -0.452201 -1.402372 1.123711
O -0.546803 -0.599248 2.068368
O -1.296095 -2.004758 0.429722
Na 1.153791 -2.319058 -0.740840
Na -2.219520 -1.124348 -1.568445
Na 1.636733 0.337105 2.106755
Na -2.873057 -0.441304 1.975974
Cl 0.224963 -0.165928 -2.190808
Cl 3.157849 -1.108406 0.407555
Na -0.897076 1.990549 -0.742116
Cl -3.294559 0.878149 -0.237801
Na 2.611404 0.961557 -1.200236
Cl 1.318887 2.501877 0.558171

[Na6Cl4CO2]+, isomer II
E=-3003.583755
Na -2.061854 -1.682726 -0.131847
C -2.112863 -0.349937 2.246572
Na -4.362078 0.933528 0.735802
Cl -3.049358 0.465956 -1.488026
Na -0.475434 1.024537 -1.892856
Cl 1.098354 2.303918 -0.148574
Na 3.652272 2.077335 0.310240
Cl 3.744351 -0.497882 0.867338
Na 2.996310 -2.194181 -0.966955

Cl 0.460932 -1.522239 -1.259405
Na 1.010350 -0.119476 1.168230
O -0.996136 -0.749751 1.871207
O -3.253961 -0.634668 1.823683

[Na6Cl4CO2]+, isomer III
E=-3003.570048
Na 4.938458 -0.973320 -0.437668
Cl 4.451643 1.361018 0.437157
Na 1.929157 1.880650 0.101423
Cl -0.593388 2.680844 0.010921
Na -3.150842 3.126900 -0.076890
Cl -3.964937 0.633752 -0.166373
Na -4.310840 -1.929894 0.121947
Cl -1.798272 -2.666947 0.224527
Na 0.779101 -3.007162 0.282940
C 2.191106 -0.844615 -0.500054
Na -1.234226 0.035988 -0.115559
O 2.799135 -1.934049 -0.396483
O 1.048202 -0.509006 -0.133984

[Na7Cl5C2HO3]+, isomer I
E=-3740.218175
C -4.007300 -0.150363 -0.239788
C -2.656046 -0.324351 -0.958457
O -2.386311 -1.482689 -1.370382
O -4.147449 0.654923 0.657869
O -1.902616 0.688415 -1.044498
Na -1.982064 1.345978 1.420722
Na -1.469571 -3.040055 0.036232
Na -0.674071 2.529637 -1.554217
Na -0.150902 -0.789673 -1.908766
Cl -1.226071 -1.212316 1.965297
Cl -0.182575 3.252010 0.915252
Na 1.407440 -1.618865 1.940859
Na 3.260361 -0.933542 -1.073493
Cl 3.425534 -0.003821 1.394778
Cl 1.199051 -2.733878 -0.632018
H -4.821890 -0.826702 -0.553822
Na 2.237797 2.221418 0.572849
Cl 1.688420 1.164237 -1.994879

[Na7Cl5CO2]+, isomer I
E=-3626.243084
C -1.738266 -1.787145 -1.309495
O -2.557945 -1.385756 -0.473969
O -0.812071 -2.621310 -1.312913
Na -2.666423 0.779488 -1.542020
Na -2.431167 -0.583921 1.736541
Na 0.651654 -1.305933 -2.547156
Na 0.543043 -3.079561 0.461877
Cl -2.699143 1.921576 0.830920
Cl 0.017502 1.323214 -2.134973
Na -0.286376 2.820480 0.282098
Na 2.039810 -0.143198 1.980118
Cl 2.173175 2.370086 1.147737
Cl -0.102260 -1.613684 2.541610
Na 2.633226 1.314370 -1.197033
Cl 2.497095 -1.356462 -0.548040

[Na8Cl6C2HO3]+, isomer I
E=-4362.866422

C 4.322437 -1.545678 0.992957
O 4.965490 -0.532905 0.765100
C 2.804516 -1.576539 0.758819
O 2.144037 -0.558643 1.136443
O 2.407305 -2.564814 0.115689
Na 1.295815 0.900299 2.632618
Na 1.252379 -2.433661 -1.782542
Na 3.465196 1.053828 -0.179573
Na 0.097001 0.129337 -0.059493
Cl 1.452728 2.671574 0.588090
Cl 1.891800 0.206684 -2.265352
Na -3.284089 -0.526189 -1.844719
Cl -4.659230 -0.504712 0.432846
Na -2.651260 -1.970533 1.395535
Na -3.132804 1.596637 1.067211
Cl -1.289722 -2.166077 -0.972145
Cl -1.250883 0.142682 2.423956
H 4.815208 -2.491371 1.277972
Na 0.568347 2.601449 -1.990743
Cl -1.875753 1.744715 -1.357262

[Na8Cl6CO2]+, isomer I
E=-4248.900641
C -2.218570 1.354793 1.988996
O -1.992925 0.121098 1.948167
O -2.945822 2.046664 1.256846
Na -1.245488 -1.938195 2.438822
Na -1.770028 3.066631 -0.405084
Na -3.620404 -0.104523 0.122209
Na -0.366185 -0.201118 0.031382
Cl -2.174152 -2.438561 -0.093688
Cl -2.168186 0.847411 -1.950251
Na 2.449141 1.261751 -1.754498
Cl 4.296766 0.437528 -0.018382
Na 2.552139 1.155657 1.865724
Na 3.003281 -1.892966 -0.077013
Cl 0.784647 2.351997 0.141374
Cl 1.328083 -1.298015 2.001061
Na -1.339718 -1.683859 -2.581652
Cl 1.258210 -1.180831 -2.057342

[Na9Cl7C2HO3]+, isomer I
E=-4985.503075
C 2.102451 1.297842 -3.249169
C 0.865511 0.453163 -2.920015
O -0.230477 1.059749 -3.011086
O 3.067886 1.303072 -2.506342
O 1.050585 -0.729019 -2.505460
Na 2.589643 0.018450 -0.569740
Na 1.051561 -2.823803 -1.654853
Na -1.408140 2.380604 -1.375910
Na -1.413465 -0.655926 -1.878953
Cl 0.030733 0.516191 0.308679
Cl 2.399061 -2.460732 0.707086
Na 1.170621 2.737188 1.575820
Cl -1.185462 3.782289 0.931309
Na -2.385662 1.402620 1.580043
Cl -2.465307 -1.052735 2.735105
Na -0.258551 -1.957370 1.525452
Na -3.652301 -1.778484 0.475395
Cl -3.524133 0.627724 -0.851239
Cl -1.471295 -3.071383 -0.719557

H 2.046507 1.953265 -4.135894
Cl 3.472674 1.314483 1.686995
Na 3.914037 -1.112275 2.482900

[Na9Cl7CO2]+, isomer I
E=-4871.535761
C 0.387587 2.042084 2.307776
O 1.419310 2.703454 2.108536
O 0.226928 0.800610 2.411803
Na -1.640908 0.538110 0.830381
Na 0.265135 -1.383519 3.101631
Na 1.590760 3.544228 -0.022914
Na 2.674968 0.721687 1.690737
Cl -0.650682 2.509554 -1.010498
Cl -1.687630 -2.234312 1.586035
Na -3.082991 1.603494 -1.819560
Cl -1.742294 -0.836930 -1.752654
Na 0.733132 0.428721 -2.263174
Cl 1.748928 -2.047160 -2.584135
Na 0.150467 -2.551259 -0.484259
Na 3.670971 -1.345252 -1.021462
Cl 3.054291 1.359157 -0.881441
Cl 2.300053 -1.902399 1.343332
Cl -4.311025 0.864466 0.388185
Na -3.779111 -1.683065 -0.058597

[Na10Cl8C2HO3]+, isomer I
E=-5608.156315
C -1.345126 3.788885 -1.455676
O -2.539024 3.848015 -1.656315
C -0.592028 2.452279 -1.244977
O 0.597184 2.574065 -0.821743
O -1.181456 1.376323 -1.523428
Na -3.517786 1.928957 -0.790628
Na 2.916554 2.914278 -0.534992
Na -1.485792 -1.058956 -0.971212
Na 1.064167 0.275388 -1.441972
Cl -4.162293 -0.650615 -0.449220
Cl 0.320460 -0.402741 1.161405
Na 0.271623 2.310024 1.690439
Na -2.409866 -0.483227 1.851444
Cl -2.021028 -3.145679 0.978718
Cl -2.396276 2.216183 1.655488
Na 0.725890 -3.122289 0.616704
Cl 3.358789 -2.859875 0.895118
Na 3.202860 -0.197434 1.394867
Na 3.420151 -2.253120 -1.671001
Cl 0.673654 -2.307190 -2.087745
Cl 3.697869 0.459103 -1.355726
H -0.716764 4.696491 -1.412518
Cl 2.886859 2.440150 2.032631
Na -4.438761 -3.247204 -0.006652

[Na10Cl8C2HO3]+, isomer II
E=-5608.139436
C -0.950836 0.349862 -1.370047
O -1.880674 -0.069119 -2.037600
C 0.363951 -0.417569 -1.248603
O 0.982577 -0.202882 -0.178907
O 0.699132 -1.232709 -2.151303
Na -1.449292 -2.505874 -1.901451
Na -3.816595 0.348082 -0.623413

Na 0.131151 -0.382021 1.958948
Na 2.499247 1.342675 0.377766
Na 2.199296 -2.119636 0.026436
Cl 4.328594 -0.549527 -0.990402
Na 5.337139 -0.805368 1.453140
Cl -2.633424 -0.150640 1.924662
Cl -3.993748 -2.365709 -1.036452
Cl 2.912897 -0.713138 2.386901
Na -2.949032 -2.777824 1.345323
Cl -0.338307 -3.017583 0.651212
H -1.051121 1.239314 -0.732550
Na 2.552521 0.083984 -2.946352
Cl 0.354831 2.501936 1.766842
Na -0.383315 3.599888 -0.660065
Cl 1.571750 2.413022 -2.079288
Cl -3.010067 3.001745 -0.367049
Na -2.312826 2.503624 2.154004

[Na10Cl8C2HO3]+, isomer III
E=-5608.129879
C -0.791756 1.047759 -0.969886
C 0.551305 0.325704 -1.073078
O 1.095253 0.061773 0.026857
O 0.969523 0.012580 -2.223493
O -1.606262 1.031842 -1.883325
H -1.040206 1.439942 0.023097
Na -1.983289 0.553737 2.867065
Na -2.317991 -2.856380 -0.134880
Na -1.008761 -1.184502 -2.932704
Cl -3.266550 2.188380 1.166101
Na -0.953206 3.656614 1.091762
Cl 0.797846 3.452114 -0.975217
Cl 0.411928 1.920924 2.739761
Na 2.424874 1.982146 0.719456
Cl 4.131114 0.950349 -1.085265
Na 2.693059 -1.443630 -0.927462
Na 1.165436 -0.659143 2.195032
Cl 2.984737 -2.463865 1.624186
Na 0.791036 -3.784152 1.287186
Cl -1.248153 -2.028887 2.236228
Cl 0.156426 -3.081723 -1.252639
Na -3.865788 1.011443 -1.190484
Cl -3.569718 -1.514082 -1.990559
Na 2.332369 1.930700 -2.701312

[Na10Cl8CO2]+, isomer I
E=-5494.185759
C 0.257184 2.321598 1.685020
O -0.942303 2.423948 1.371513
O 0.955406 1.336739 2.011941
Na 2.965619 2.555470 1.296289
Na -3.279815 2.524685 0.986050
Na 1.688369 -0.894789 1.084416
Na -1.126594 -0.031307 1.709831
Cl 4.199374 0.202549 0.758253
Cl -0.127716 0.004491 -0.936280
Na -0.443522 2.782024 -1.130804
Na 2.560355 0.392595 -1.621726
Cl 2.628700 -2.392142 -1.187495
Cl 2.220438 3.032016 -1.195929
Na -0.111864 -2.808242 -1.050784
Cl -2.724234 -2.856601 -1.539322

Na -2.958822 -0.157570 -1.472876
Na -3.048902 -2.810603 1.076251
Cl -0.354555 -2.598922 1.758656
Cl -3.723291 -0.148289 1.282747
Cl -3.032441 2.561107 -1.613686
Na 5.017483 -2.160140 -0.125354

[Na11Cl9C2HO3]+, isomer I
E=-6230.809015
Na -2.250871 1.011881 0.247600
Cl 0.079683 0.095492 -1.153206
Cl -1.287908 0.687851 2.794162
Na -2.116039 -1.856698 2.443628
Cl 0.273199 -2.851073 1.488574
Na -0.340492 3.199054 2.645126
Cl 2.048670 2.499176 1.762613
Cl -3.840218 1.586685 -1.951079
Na -2.376011 -0.550598 -2.352986
Na -3.251311 4.073479 -1.623874
C -2.852618 -2.421580 -0.362921
Cl -1.303738 3.715349 0.126599
Na 1.119813 -0.166019 1.575687
Cl 3.688142 -0.862631 0.783581
Na 4.340288 1.739861 0.514559
Cl 3.440722 2.069412 -1.970986
Na 1.116167 2.705399 -0.830867
Na 2.819304 -0.553992 -1.864409
Cl 2.305480 -3.206865 -2.088693
Na -0.109728 -2.750989 -1.212570
Na 2.812892 -3.381057 0.530124
C -3.447045 -3.608340 0.425695
O -2.410670 -2.706363 -1.513755
O -2.828377 -1.294195 0.192248
O -3.468194 -3.625171 1.640484
H -3.816753 -4.455928 -0.177253

[Na11Cl9C2HO3]+, isomer II
E=-6230.776607
C -0.263914 -0.317683 -0.489939
O -1.459302 -0.056667 -0.526871
C 0.730161 0.338408 -1.473349
O 1.946133 0.154576 -1.209663
O 0.248066 1.030271 -2.409540
Na -1.371994 -0.631480 -3.153885
Na -3.449286 1.622275 -0.879233
Na 3.878877 1.405060 -1.686332
Na 2.854878 -2.057146 -1.211063
Na -3.648008 -1.427352 -0.029243
Na 2.864716 0.196490 1.041433
Na -1.913602 0.627204 1.882250
Cl -4.475143 0.667550 1.366374
Na 0.379215 3.176274 -1.569580
Cl 2.583082 3.059790 0.039797
Cl 5.006335 -0.591962 -0.501655
Na 0.733261 3.359411 1.990278
Cl -1.432764 3.101789 0.386436
Cl -3.895086 -0.447976 -2.572837
Cl 2.095087 -2.579423 1.390683
Na 0.446643 -1.848501 3.368017
Cl -1.786885 -2.298289 1.892174
Cl 0.709941 0.775332 2.763371
Cl 0.255088 -2.649741 -2.160959

Na -0.125653 -3.679031 0.283544
H 0.169702 -0.955233 0.292748

[Na11Cl9C2HO3]+, isomer III
E=-6230.759845
C -0.931809 -0.470608 -0.273661
C 0.196842 -1.446883 0.084271
O -0.159814 -2.579859 0.488136
O -0.700821 0.712533 -0.460480
O 1.366976 -1.013696 -0.090548
Na 2.825875 -1.228033 1.659915
Na -1.733800 -1.815691 2.302193
Na -1.076586 0.668199 -3.009060
Cl -0.329678 -2.011275 -2.880198
Na 2.184037 -2.390385 -1.725708
Cl 4.425604 -2.542754 -0.269609
Na 5.728903 -0.292335 -0.163831
Cl -2.962153 0.762892 2.072201
Na -0.628813 1.803048 3.083292
Cl 0.564653 -0.534283 3.136687
Na -2.279126 2.407345 -0.049500
Cl -0.019676 3.557793 1.056745
Na 1.040475 4.629146 -1.134298
Cl 0.932948 2.505790 -2.617701
Na 1.527500 1.383173 -0.118312
Cl 3.976255 1.228474 0.988955
H -1.955206 -0.871483 -0.337685
Na -4.595641 -0.354638 0.128232
Cl -3.678243 -2.806133 0.618551
Cl -3.593216 1.046999 -1.952447
Na -1.726106 -3.455550 -1.029920

[Na11Cl9CO2]+, isomer I
E=-6116.829379
Na -2.187086 0.179653 -0.012034
Cl 0.281985 0.013243 -1.111496
Cl -1.289679 0.147863 2.610566
Na -1.273528 -2.534683 2.431277
Cl 1.228636 -2.861621 1.653465
Na -0.900424 2.795533 2.378151
Cl 1.619677 2.771083 1.602923
C -5.566817 0.326405 -0.897971
Na -4.724589 -2.221623 -1.156521
Na -4.301179 2.669463 -1.280699
Cl -2.384045 -2.668403 -0.074399
Cl -1.990051 3.041614 -0.130821
Na 1.372077 -0.043996 1.514937
Cl 4.008357 -0.232658 0.607575
Na 4.011104 2.436431 0.307958
Cl 3.090953 2.544631 -2.170782
Na 0.684212 2.736244 -1.017879
Na 2.943741 -0.180250 -2.010171
Cl 2.706149 -2.901221 -2.123332
Na 0.299220 -2.739383 -0.966715
Na 3.636606 -2.879998 0.353153
O -4.316115 0.365065 -1.112491
O -6.356945 -0.599662 -0.793892
